# Activity Control of a Synthetic Transporter by Photodynamic Modulation of Membrane Mobility and Incorporation

**DOI:** 10.1021/jacs.4c10952

**Published:** 2024-11-01

**Authors:** Jasper E. Bos, Maxime A. Siegler, Sander J. Wezenberg

**Affiliations:** † Leiden Institute of Chemistry, Leiden University, Einsteinweg 55, 2333 CC Leiden, The Netherlands; ‡ Department of Chemistry, Johns Hopkins University, 3400 N. Charles St., Baltimore, Maryland 21218, United States

## Abstract

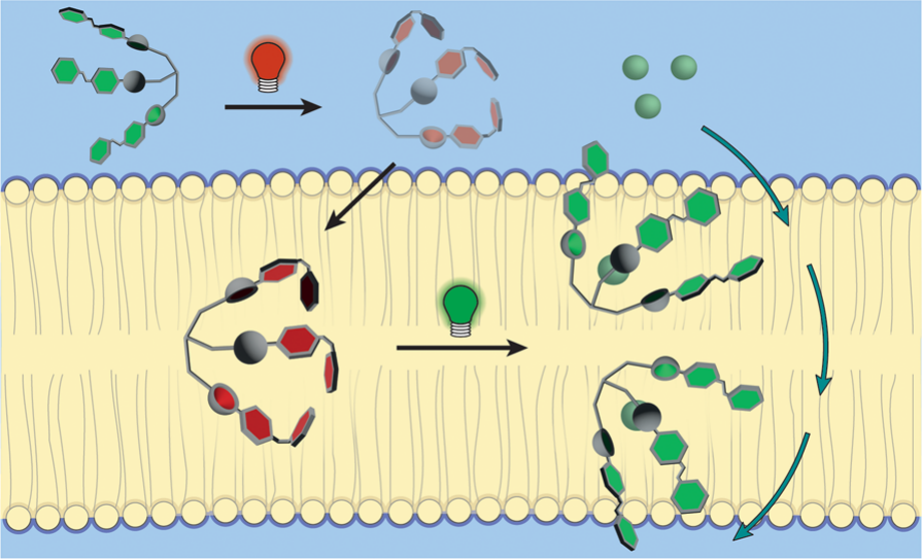

Artificial transmembrane transport systems are receiving a great deal of attention for their potential therapeutic application. A major challenge is to switch their activity in response to environmental stimuli, which has been achieved mostly by modulating the binding affinity. We demonstrate here that the activity of a synthetic anion transporter can be controlled through changes in the membrane mobility and incorporation. The transporters—equipped with azobenzene photoswitches—poorly incorporate into the bilayer membrane as their thermally stable (*E*,*E*,*E*)-isomers, but incorporation is triggered by UV irradiation to give the (*Z*)-containing isomers. The latter isomers, however, are found to have a lower mobility and are therefore the least active transporters. This opposite effect of *E*-*Z* isomerization on transport capability offers unique photocontrol as is demonstrated by *in situ* irradiation studies during the used transport assays. These results help to understand the behavior of artificial transporters in a bilayer and are highly important to future designs, with new modes of biological activity and with the possibility to direct motion, which may be crucial toward achieving active transport.

## Introduction

Membrane-embedded proteins are essential for many important cellular functions.^1^ Among them are transporters, which mediate the passage of ions and solutes across the lipid bilayer, and are involved in processes such as signal transduction, ion homeostasis, and regulation of osmotic pressure. Many of these proteins are able to switch between high- and low-affinity modes—as a means to control their transport activity—in response to environmental stimuli.^2^ Other proteins are known to undergo changes in mobility as a way to control their biological function. Such mobility changes are often caused by local fluctuations in lipid composition and can also be due to protein aggregation and/or shape changes.^3^

Over the past decades, much effort has been devoted to the development of artificial anion transport systems,^4^ owing to their therapeutic potential.^5^ It is of interest to make such systems capable of responding to environmental stimuli,^6^ for example, to allow local (de)activation. The predominant strategy to achieve this is based on (dynamic) control of binding affinity using pH change,^7^ light,^8^ or redox agents,^9^ where light has the benefit that it is noninvasive. Another frequently used approach is to influence membrane partitioning via light- or chemically cleavable water-soluble groups that are installed to known anion transporters.^10^ In addition, the group of Langton recently showed that photocleavage of a membrane-anchoring group (i.e., long alkyl chains) leads to activation of anion transport.^11^ Cleavage of these groups, however, is an irreversible process. So far, to the best of our knowledge, there have been no designs of photodynamic transporters that deliberately target control of membrane mobility and incorporation, while such control could enable directed motion and would offer an alternative approach to alter biological properties.

To fill this gap, we considered functionalizing tren-based tris-thiourea transporters [tren = tris(2-aminoethyl)amine], which were previously developed by Gale and co-workers^12^ (and for which the transport mechanism is well understood) with azobenzene photoswitches. In such a transporter, the impact of *E*/*Z* isomerization on the binding properties would be minimal. Conversely, owing to the large change in dipole moment of azobenzene upon isomerization, and resultantly a large difference in solubility between the *E* and the *Z*-isomer,^13^ membrane mobility and incorporation were expected to be largely influenced. An additional benefit of the tren-based scaffold is that it allows appendage of up to three azobenzene groups to enhance these expected effects.

**Scheme 1:**
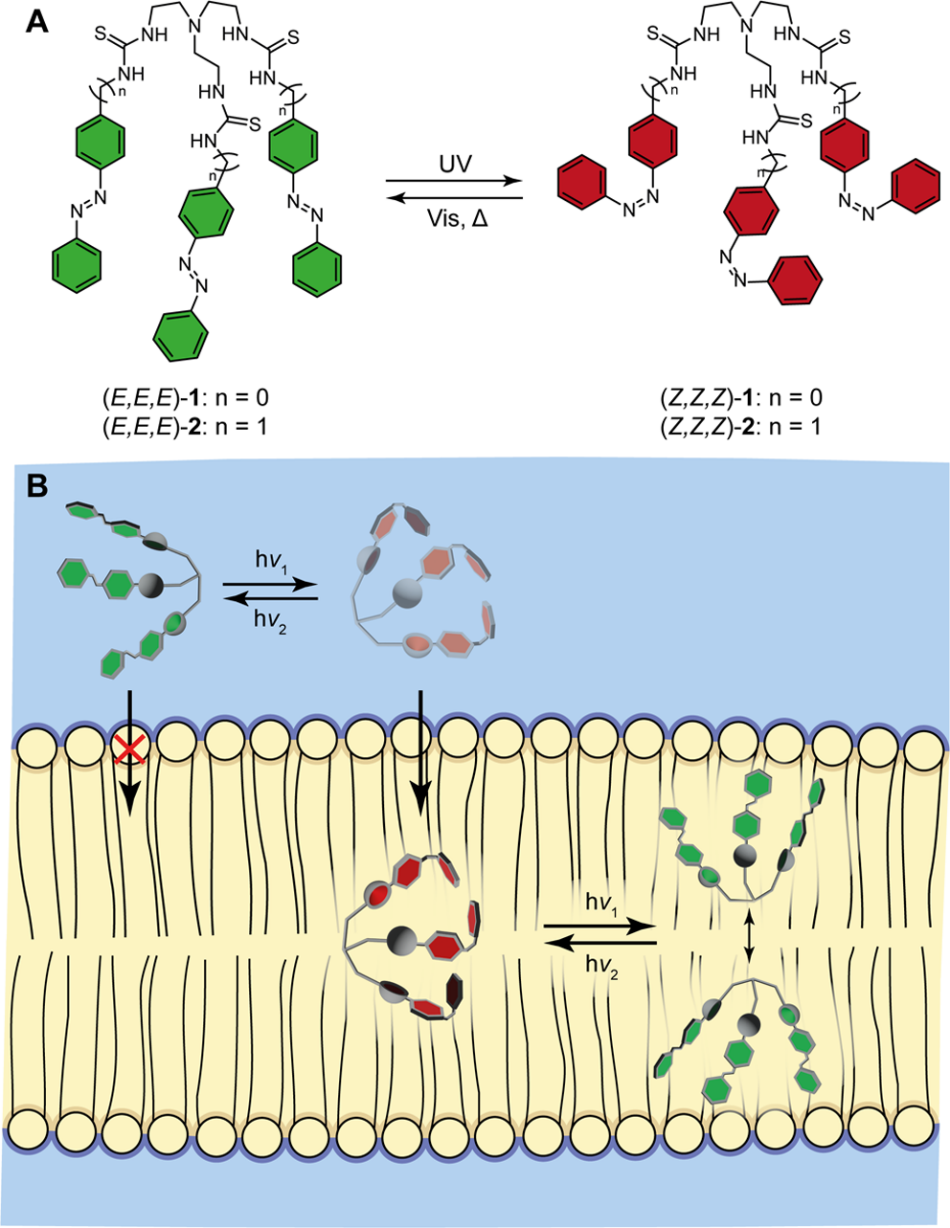
Photoisomerization of Azobenzene-Based Tris-Thioureas **1** and **2** (A) and Schematic Representation of the Photocontrol over Membrane Incorporation and Mobility (B)

Herein, we describe the azobenzene-appended tren-based tris-thiourea transporters **1** and **2** (Scheme 1A), whose isomers can be interconverted by UV and visible light. The half-lives of the photogenerated, metastable state of these two compounds are different. That is, the thiourea substitution in the benzylic position in **2** is known to afford higher thermal stability of azobenzene as compared to the direct substitution in **1**. We show here that the photogenerated *Z*-containing isomers of these transporters are better incorporated into the lipid bilayer membrane, while their mobility—and with that their transport activity—is lower than the corresponding (*E,E,E*)-isomers (Scheme 1B). These opposing effects of isomerization on the transport capability are shown to give unprecedented control of the transport process. Furthermore, the observed dynamic modulation of membrane mobility will be key toward directed motion and to achieve active transport in the future.

## Results and Discussion

### Synthesis and Isomerization Behavior

**Figure 1:**
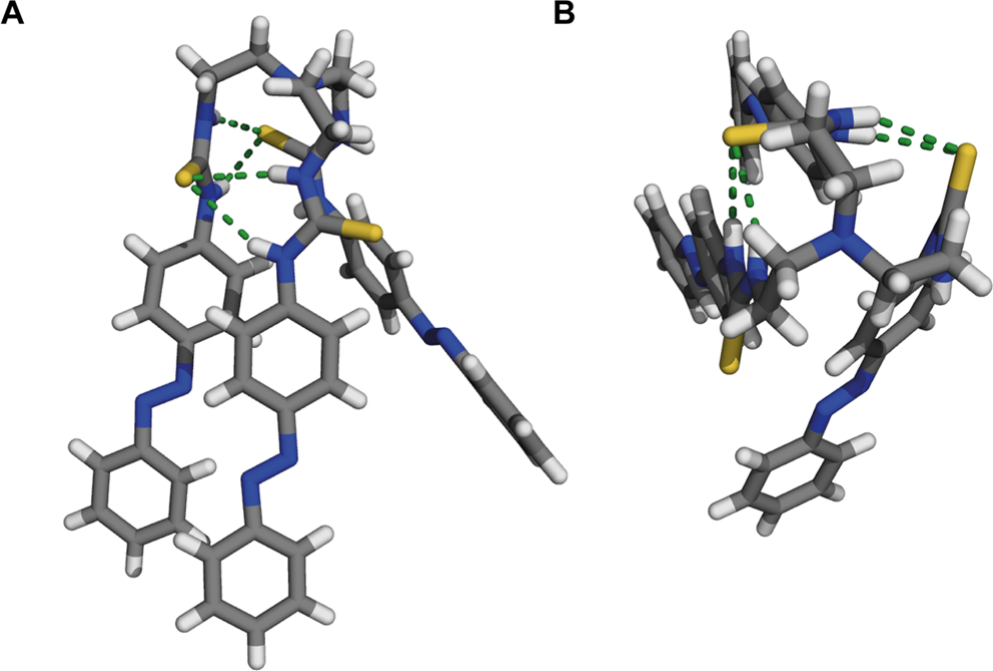
Side view (A) and top view (B) of (*E,E,E*)-**1** as found in the crystal structure, shown in stick representation. Disorder in the azobenzene moieties was omitted for clarity.

Transporters **1** and **2** were synthesized in one step by reacting previously reported tris(2-isothiocyanatoethyl)amine^14^ with commercially available 4-aminoazobenzene and known 4-aminomethylazobenzene,^15^ respectively. After purification by column chromatography, followed by recrystallization, the compounds were isolated exclusively as their (*E,E,E*)-isomers (see the Supporting Information for synthetic details and characterization). For compound **1**, single crystals suitable for X-ray structure determination were obtained from a mixture of CHCl_3_/MeOH. The solid-state structure, depicted in Figure 1, further confirms the isolation of the (*E,E,E*)-isomer. Interestingly, it shows involvement of two of the thiourea groups in intramolecular hydrogen bonding with the sulfur atom of a neighboring thiourea group [N(H) ··· S distance: 3.5585(12)–3.3622(13) Å].

**Figure 2:**
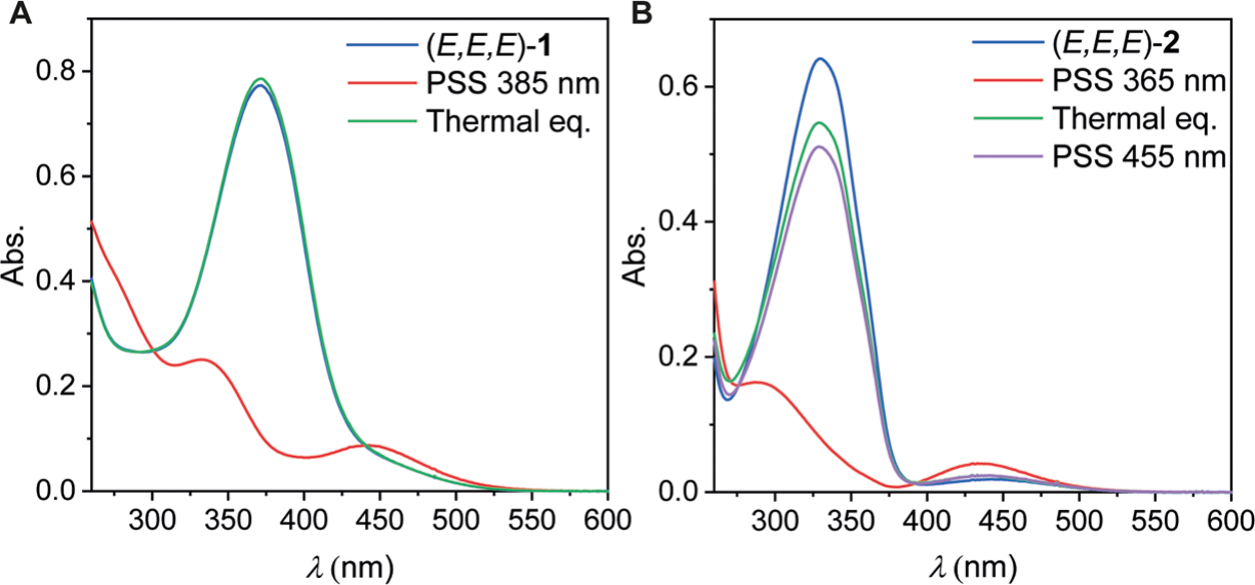
UV–vis spectral changes measured at 293 K of (*E,E,E*)-**1** (A) and (*E,E,E*)-**2** (B) in DMSO/MeCN (1:1 v/v, 1 × 10^–5^m) upon irradiation with 385, 365 or 455 nm light, as well as upon thermal equilibration.

Next, the photoswitching properties of the obtained compounds were investigated with UV–vis and ^1^H NMR spectroscopy. Early in our studies, we noted that mixing MeCN with DMSO increased the thermal stability of the photogenerated isomers of **1**, when compared to DMSO alone, likely due to aggregation effects.^16^ To avoid quick thermal decay, the switching behavior was characterized in DMSO/MeCN (1:1 v/v). In this mixture, tren-based tris-thiourea (*E,E,E*)-**1** displayed an absorption maximum around 375 nm (Figure 2A). Upon irradiation with 385 nm light, this maximum decreased, while a new absorption maximum appeared at λ = 450 nm, characteristic of *E*-to-*Z* isomerization. An isosbestic point was observed at around λ = 301 and 440 nm (Figure S5), which indicates independent switching of the azobenzene units. In the dark, the original spectrum of the (*E*,*E*,*E*)-isomer was regained as a result of thermally promoted back isomerization, and a half-life of 49 min at 293 K was determined for the photogenerated isomers (Figure S6).

Compared to (*E,E,E*)-**1**, the absorption of (*E,E,E*)-**2** was blue-shifted and a maximum was observed around λ = 330 nm (Figure 2B). Therefore, a shorter wavelength of light, i.e., 365 nm, was used here to promote *E*-to-*Z* isomerization, resulting again in a decrease of the overall absorption and the appearance of a new maximum around λ = 430 nm, where isosbestic points were observed at λ = 276 and 392 nm (Figure S7). As anticipated, the thermal *Z-*to-*E* isomerization was much slower in this case than that for **1**. It was therefore followed at 333 K, and the half-life at this temperature was determined as 120 min (Figure S8). This back isomerization could be accelerated by irradiation with 455 nm light (Figures 2B and S9). In contrast to compound **1**, at thermal equilibrium, the initial spectrum of the (*E,E,E*)-isomer was not fully recovered for compound **2**, indicating that some of the *Z*-containing isomers remained present in solution. As usual for azobenzene,^13b^ also 455 nm irradiation did not give full conversion back to the (*E*,*E*,*E*)-isomer, but led to the formation of a photostationary state (PSS) mixture.

These isomerization processes were then followed by ^1^H NMR spectroscopy in DMSO-*d*_6_/MeCN-*d*_3_ (1:1 v/v) in order to determine the PSS ratios. Irradiation of solutions containing either **1** or **2** with 385 and 365 nm light, respectively, led to the appearance of new sets of signals belonging to the *Z*-azobenzene-containing isomers (Figures S16 and S17). It should be noted that no separate sets of signals were observed for the possible different photogenerated isomers [i.e., (*Z,Z,Z*), (*Z,E,E*), (*Z,Z,E*)]. As by ^1^H NMR spectroscopy these isomers could thus not be distinguished, the overall *E*/*Z* ratio for the PSS mixtures was determined only (see Table 1). Nevertheless, by assuming that each azobenzene moiety switches independently, which is supported by the observation of clear isosbestic points (*vide supra*), we calculated that the PSS_UV_ mixture for **1** and **2**, from here on denoted as *Z*_PSS_, consists of at least 70% of the (*Z,Z,Z*)-isomer and less than 5% as the (*Z,E,E*)- or (*E,E,E*)-isomers (Figure S19). Finally, similar to what was observed in the UV–vis experiments, the initial signals of the (*E*,*E*,*E*)-isomer were recovered over time in the dark. In the case of **2**, back isomerization was again also induced by irradiation with 455 nm light (see Figure S18 and see Table 1 for all PSS and thermal equilibrium ratios).

**Table 1:** Photoswitching, Chloride Binding, and Transport Properties of **1** and **2**

compound	equil. ratio (*E*/*Z*)	PSS_UV_ (*E*/*Z*)	PSS_Vis_ (*E*/*Z*)	*K*_a(*E,E,E*)_^a^ (m^–1^)	*K*_a(*Z*_pss_)_^a^ (m^–1^)	EC_50(*Z*_pss_)_^b^ (mol %)	EC_50(*E,E,E*)_^b^ (mol %)	*F* (*E*/*Z*)^d^
1	100:0	11:89	n.d.	2.7 × 10^2^	n.d.	0.010	0.086	8.6
2	91:9	7:93	77:23	2.9 × 10^2^	180	0.037	>10^c^	>270

a Determined by ^1^H NMR titrations using the tetrabutylammonium chloride salt in DMSO-*d*_6_/0.5%H_2_O; errors are estimated to be no more than 15%.

b EC_50_ is defined as the effective concentration needed to reach 50% of the maximum activity at *t* = 360 s; values are reported in transporter-to-lipid molar ratio.

c Poor transport activity prevented full Hill analysis.

d Factor of enhancement in chloride transport activity between the (*E,E,E*)-isomer and (*Z*_PSS_)-isomers (*F*_(E/Z)_ = EC_50(*E,E,E*)_/EC_50(*Z*pss)_); when EC_50_ > 10, a value of 10 was used in the calculation.

### Binding and Transport Properties

We then determined the strength of chloride binding starting with the (*E,E,E*)-isomers using ^1^H NMR titrations in DMSO-*d*_6_/0.5% H_2_O. This solvent mixture was chosen in order to compare the binding constant to other tren-based tris-thioureas reported in the literature.^12b^ A downfield shift in the thiourea NH signals was observed upon the stepwise addition of tetrabutylammonium chloride ([Bu_4_N]^+^[Cl]^−^), in addition to relatively small chemical shift changes in the aromatic and aliphatic signals (Figures S20–21). The titration data was fitted to a 1:1 binding model using HypNMR software (Figures S23–24), giving similar binding constants for (*E,E,E*)-**1** and (*E,E,E*)*-***2** (Table 1). These constants are of the same order of magnitude as those reported previously for aromatically substituted tren-based tris-thiourea compounds (e.g., *K*_a_ = 191 m^–1^ for the analogue containing phenyl instead of azobenzene groups).^12b^

We then also determined the (apparent) binding affinity of the *Z*_PSS_ mixture of **2**. It should be noted that a titration using (*Z*_PSS_)-**1** could not be performed due to fast thermal back isomerization in the solvent mixture used for titrations; however, its chloride binding affinity is expected to be similar to (*Z*_PSS_)-**2**. As also observed for the (*E*,*E*,*E*)-isomer, the addition of tetrabutylammonium chloride to (*Z*_PSS_)-**2** led to a gradual downfield shift of the thiourea NH signals, alongside small changes in the aromatic and aliphatic signals (Figure S22). Fitting to a 1:1 binding model afforded a *K*_a_ value that shows minimal difference with (*E,E,E*)-**2** (i.e., 1.6-fold, see Figure S25 and Table 1). As anticipated, photoswitching thus has only a small effect on the binding strength of the azobenzene-appended tren-based tris-thiourea transporter.

After having established that the binding affinity is minimally affected by photoswitching, we set out to investigate whether the isomers of **1** and **2** have distinct transmembrane chloride transport properties. Initial assays for screening were conducted with large unilamellar vesicles (LUVs) from 1-palmitoyl-2-oleoyl-*sn*-glycero-3-phosphocholine (POPC), loaded with the pH-sensitive dye 8-hydroxypyrene-1,3,6-trisulfonate (HPTS), and suspended in a NaCl solution buffered to pH 7.0 with HEPES.^17^ After the addition of the compounds in DMSO/MeCN (1:1 v/v) to the vesicle solution, a pH gradient was applied across the membrane through addition of a base pulse (NaOH). The ability of the compounds to dissipate this pH gradient through H^+^/Cl^–^ symport (or OH^–^/Cl^–^ antiport) was then monitored via the change in HPTS emission.

**Figure 3:**
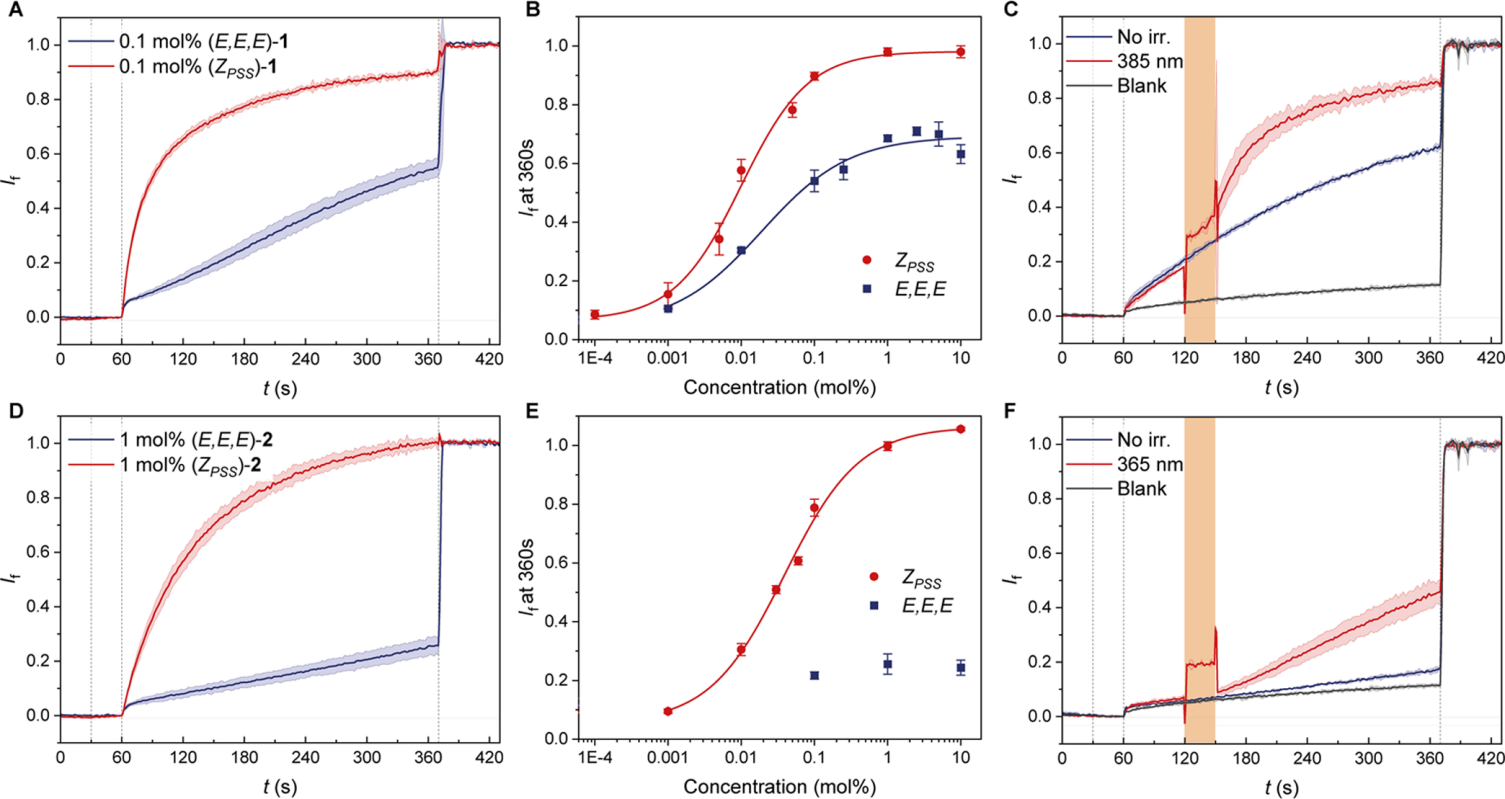
Plots of chloride efflux against time facilitated by the (*E,E,E*)-isomers (blue line) and (*Z*_PSS_)-isomers (red line) of **1** (A) and **2** (D). Hill plots of activity versus concentration of the (*E,E,E*)-isomers (blue squares) and (*Z*_PSS_)-isomers (red circles) of **1** (B) and **2** (E). Change in chloride efflux upon irradiation of 0.1 mol % (*E,E,E*)-**1** at 385 nm (C) and 1 mol % (*E,E,E*)-**2** at 365 nm (F).

In this HPTS assay, the photogenerated (*Z*_PSS_)-isomer mixture of both **1** and **2** proved to be effective in anion transport, whereas the corresponding (*E,E,E*)-isomers showed poor transport behavior when measured at the same transporter-to-lipid ratios (Figure 3A,D). The half-maximal effective concentrations (EC_50_, expressed in mol % with respect to lipids) were determined by Hill analysis (Figures 3B,E and S28–33, as well as Table 1), and showed much higher activity for the (*Z*_PSS_)-isomer mixtures than for the respective (*E,E,E*)-isomers. The largest enhancement upon irradiation was found for **2** (>270 times, see Table 1), while **1** proved to be the more active transporter. Owing to this marked difference in activity between isomers, it was possible to activate transport using either **1** or **2***in situ*. That is, addition of a solution of the (*E,E,E*)-isomer, to the vesicle solution, followed by irradiation for 30 s, led to the enhancement of chloride efflux (Figure 3C,F).

Based on these results, the logical conclusion would be that the (*Z*_PSS_)-isomers are the more active transporters, with the *in situ* activation of transport as compelling evidence. Nevertheless, it assumes that both isomers, when postadded from DMSO/MeCN to the vesicle solution, are incorporated into the bilayer to the same extent. The Hill plots obtained for the (*E,E,E*)-isomers, however, show that full efflux is never reached, also not at the highest loadings, which can be an indication of poor incorporation.^8g,17a^ Preincorporation of the transporters into the bilayer membrane, by hydrating a lipid film already containing the compounds to form the vesicles, would mitigate this issue. However, it requires the *E/Z* ratio to remain constant during the transport run; i.e., no significant thermal decay should occur. Alternatively, the PSS (*E*/*Z*) ratio could be kept constant by continuous irradiation during the experiment; however, since the HPTS dye absorbs UV light, irradiation with the wavelengths used for isomerization would additionally cause its excitation, leading to false results.

To check the thermal stability of the photogenerated *Z*-containing isomers in the lipid bilayer, we monitored the UV–vis absorption over time of vesicles that had **1** and **2** preincorporated (see Figures S10–S15). After photochemically induced isomerization, compound **2** did not show significant thermal isomerization during the measurement time frame (1 h). However, the half-life of **1** was drastically shortened (35 s) compared to what was measured in DMSO/MeCN solution (49 min). Such a difference in half-life is not uncommon for azobenzene as its thermal stability is known to be highly dependent on solvent polarity.^18^ For the HPTS assay described above, this short half-life means that the (*E,E,E*)-isomer of **1** is regained at the onset of the experiment. To determine the true activity of the (*Z*_PSS_)-isomers of **1**, it was, therefore, necessary to irradiate the vesicle solution continuously during the transport run.

As this could not be done in the HPTS assay due to excitation of the dye with the UV irradiation wavelength used to promote *E*/*Z* isomerization (*vide supra*), we turned to a cationophore-coupled osmotic response assay.^12e^ For this assay, LUVs are created containing buffered KCl, suspended in potassium gluconate (KGlu) and, to maintain the charge balance, a transporter will mediate chloride efflux only in combination with a cationophore that can transport K^+^ ions. This net KCl efflux leads to shrinkage of the vesicle, which can be followed by changes in the 90° light scattering intensity. By evaluating the difference between coupling with the cationophores valinomycin (a selective K^+^ transporter) and monensin (a K^+^/H^+^ exchanger), it can be deduced if a transporter preferentially operates as an electroneutral Cl^–^ transporter or as an H^+^/Cl^–^ (or OH^–^/Cl^–^) cotransporter.^17^

**Figure 4:**
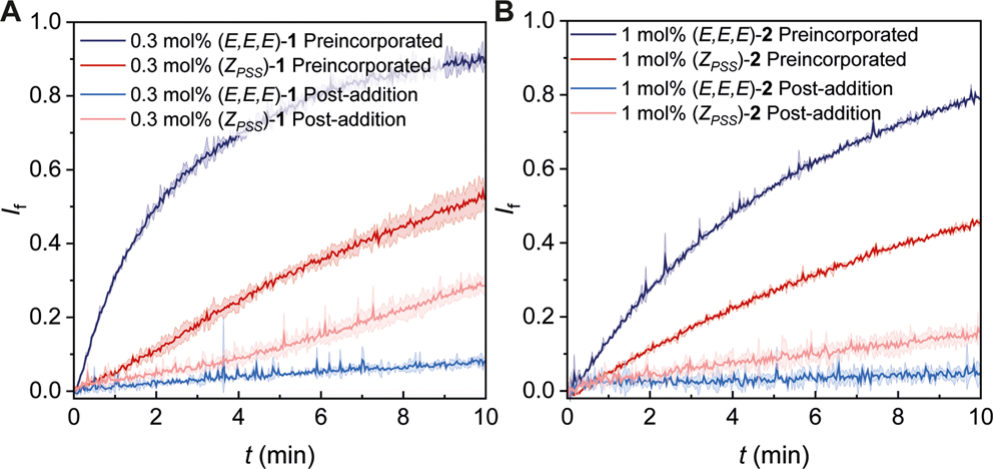
Plots of chloride efflux over time of the isomers of **1** (A) and **2** (B) facilitated by preincorporated transporter compared with postaddition from DMSO-MeCN (1:1 v/v).

We first verified that irradiation during the transport run using the osmotic assay does not lead to any undesired effects or damage to the vesicle integrity when compounds **1** and **2** are incorporated (Figures S34 and S35). Next, we addressed the potential issue with their incorporation into the membrane by comparing the (apparent) activity (in combination with valinomycin) between preincorporation and postaddition from DMSO/MeCN (1:1 v/v) at the same transporter-to-lipid ratio (Figure 4). Upon postaddition, as also observed in the HPTS assay, the (*Z*_PSS_)-isomers showed higher activity than the respective (*E,E,E*)-isomers. Yet, when preincorporated, the activity of both the (*Z*_PSS_)-isomers and (*E,E,E*)-isomers was enhanced. Strikingly, the difference in activity between these isomers was reversed compared to the postaddition studies, i.e., now the (*E,E,E*)-isomers turned out to be the most active transporters. This result clearly shows that the isomers of **1** and **2** have distinct membrane incorporation ability. In fact, the low transport rate observed for the (*E,E,E*)-isomers upon postaddition from organic solution must be due to very poor membrane incorporation.

It has been shown previously that the incorporation ability of a transporter relates to its lipophilicity, with more lipophilic transporters incorporating less well into the membrane.^19^ To gain insight into the relative lipophilicity of the isomers of **2**, we determined their retention time on a C_18_ RP-HPLC column (Table S1 and Figures S26–S27). Here, (*E,E,E*)-**2** proved to be the most lipophilic isomer, showing a large difference in lipophilicity with the (*Z,Z,Z*)-isomer. Lipophilicity thus seems to be the major factor explaining the difference in membrane incorporation, although potential aggregation of the (*E*,*E*,*E*)-isomer could also limit its incorporation ability.

Preincorporation into the bilayer thus reveals that these (*E,E,E*)-isomers are actually more active transporters than the (*Z*_PSS_)-isomers. It is important to note that, without such preincorporation studies, the inherent transport ability of the (*E,E,E*)-isomers would have been overlooked and that the activity changes upon photoswitching would have been misinterpreted. To summarize, irradiation with UV light [to generate the (*Z*_PSS_)-isomers] triggers membrane incorporation but, on the other hand, switches the transporters to a less active state. Such an opposite effect on transport capability upon isomerization has, to our best knowledge, not been reported for photoactive transporters and enables a new level of control over transport (*vide infra*).

**Figure 5:**
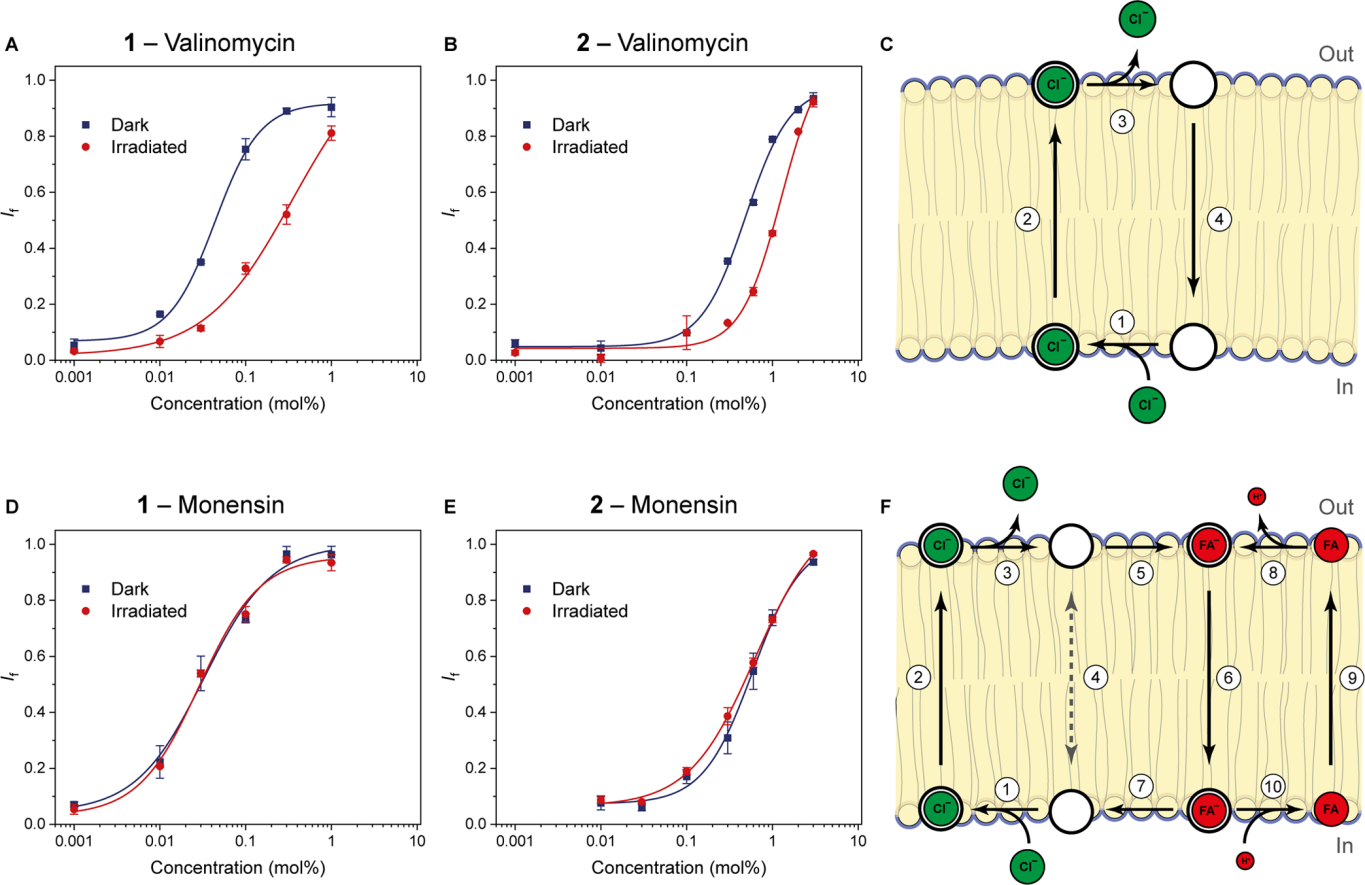
Hill plots generated using the isomers of **1** and **2** in combination with valinomycin (A, B) and the corresponding mechanism (C), and in combination with monensin (D, E) and the corresponding mechanism (F).

To quantify the difference in inherent transport activity and to gain insight into the preferred mechanism of transport, we again performed concentration-dependent (Hill) analysis, now using the osmotic assay and vesicles in which the transporters were preincorporated in the bilayer. When promoting operation as an electrogenic Cl^–^ transporter through coupling with valinomycin, the (*E,E,E*)-isomers of **1** and **2** turned out to be up to 5.4 times more active than the (*Z*_pss_)-isomer mixture (Figures 5A,B and S36–S47, and Table 2). Surprisingly, when coupled with monensin allowing H^+^/Cl^–^ symport (or OH^–^/Cl^–^ antiport), virtually no difference in activity was observed as becomes very clear when comparing the concentration-dependent curves in Figure 5D,E.

**Table 2:** Transport Properties as Determined Using the Osmotic Assay of **1** and **2**

compound	EC_50(*Z*_pss_)_^a^ Vln (mol %)	EC_50(*E*)_^a^ Vln (mol %)	*F* (*Z*/*E*)^b^	EC_50(*Z*_pss_)_^a^ Mon (mol %)	EC_50(*E*)_^a^ Mon (mol %)	*F* (*Z*/*E*)^b^
**1**	0.27	0.049	5.44	0.031	0.033	0.95
**2**	1.13	0.50	2.25	0.51	0.57	0.89

a EC_50_ defined as the effective concentration needed to reach 50% of the maximal activity at *t* = 658 s; values reported in transporter to lipid molar ratio.

b Factor of enhancement in chloride transport activity between the (*Z*_PSS_)-isomer and (*E*)-isomers (*F*_(*Z*/*E*)_ = EC_50(*Z*pss)_/EC_50(*E*)_).

This remarkable observation can be understood by taking a closer look at the rate-limiting steps in these two different transport mechanisms (Figure 5C,F). In the Cl^–^ uniport, after binding an anion at the inner leaflet of the membrane, the complexed transporter moves to the outer leaflet where the anion is released. The free transporter subsequently has to move back to the inner leaflet to pick up another anion and repeat the same cycle (Steps 1–4 in Figure 5C). For H^+^/Cl^–^ symport (or OH^–^/Cl^–^ antiport), the initial step is the same; however, additionally, the transport of a proton needs to be facilitated. For these types of transporters based on the tren scaffold, this proton transport is known to occur either through thiourea (de)protonation or by a fatty acid-assisted pathway.^12e^ In the case of the latter, fatty acids present in the lipid bilayer cannot repetitively transfer protons on their own, as in their carboxylate form (after deprotonation) they are not able to diffuse through the bilayer. Nevertheless, when a transporter binds the carboxylate headgroup, it can mediate the diffusion (flip-flop) of the fatty acid, resulting in net proton transport (Steps 5–10 in Figure 5F). Fatty acids are present as impurities in commercially available POPC,^20^ and several tren-based anion transporters have been shown to be most efficient when operating through such a fatty acid-assisted pathway.^12k^ These transporters were shown to not diffuse well through the membrane when in unbound form, yet, binding with a fatty acid gives a complex that has increased interactions with the phospholipid tails and therefore diffuses easier.^12k^ Hence, we investigated the influence of fatty acid-assisted proton transport for (*E,E,E*)-**1** and (*E,E,E*)-**2**, by either removal of fatty acids using BSA treatment or external addition of oleic acid (Figures S48–51). As can be expected based on the proposed mechanism, fatty acid concentration-dependent activity was observed when coupling to monensin but not when using valinomycin. Furthermore, the use of monensin and large amounts of fatty acid resulted in the highest transport activity, which reveals that during H^+^/Cl^–^ symport diffusion and formation of the fatty acid-transporter complex across the membrane is rate-limiting (steps 5–7).

Coming back to the Cl^–^ uniport process, here the transporter needs to move back to the inner leaflet in the unbound form. Since less activity was observed as compared to the fatty acid-assisted pathway described above, diffusion of the free transporter through the membrane must be the rate-limiting step (step 4). As in this case the (*Z*_PSS_)-isomers showed less activity than the (*E,E,E*)-isomers, and diffusion of their unbound form is rate limiting, it can be concluded that the former isomers have lower mobility in the bilayer than the latter isomers. The observed activity difference upon isomerization can thus be ascribed to a mobility change. To the best of our knowledge, this represents the first example in which reversible control over the mobility of a transporter is demonstrated.

### Single- and Dual-Wavelength Control

**Figure 6:**
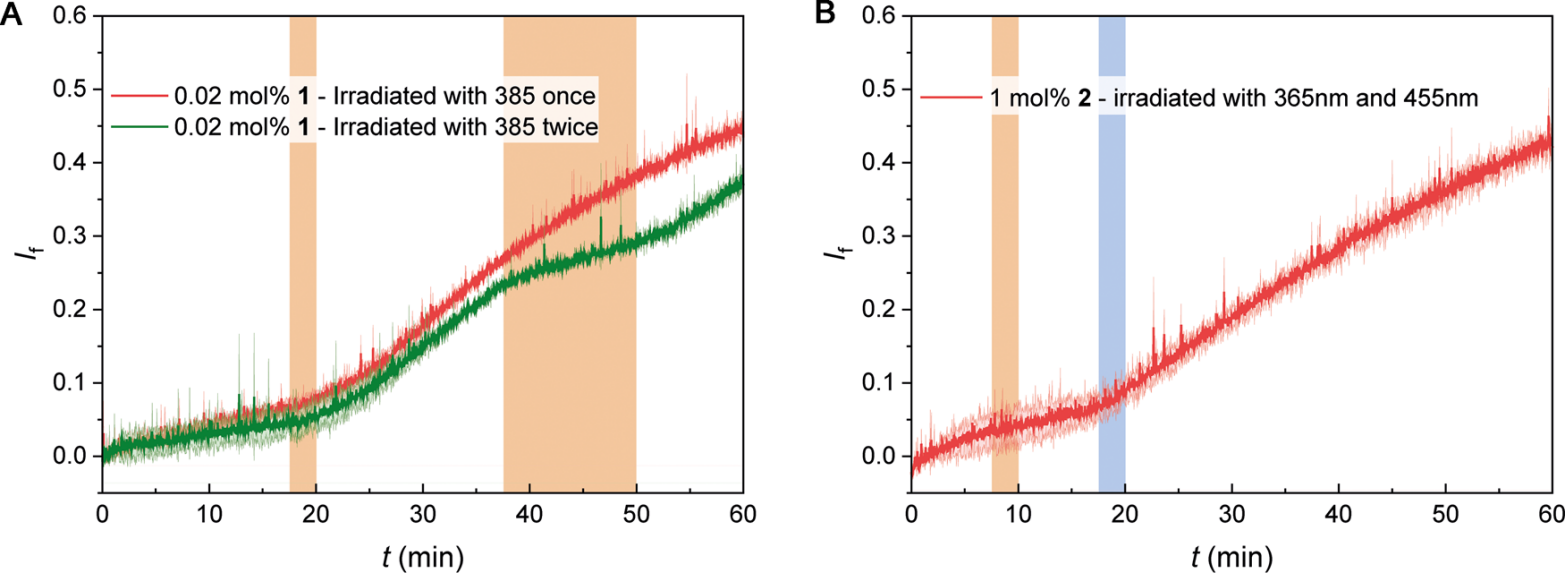
Plots of electrogenic Cl^–^ transport against time facilitated by 0.02 mol % (*E,E,E*)-**1** (A) and 1 mol % dark-adapted **2** (B) in combination with valinomycin, and activation by *in situ* irradiation. The shaded area denotes the irradiation time span using the mentioned wavelengths. For control experiments without irradiation or only a single irradiation step, see Figures S52 and S53.

While the (*E,E,E*)-isomers are thus the more active transporters owing to their higher mobility, they do not incorporate well into the bilayer. Therefore, no transport is observed once they have been postadded to the vesicle solution. Yet, their incorporation into the membrane can be triggered by UV irradiation to give the (*Z*_PSS_)-isomers, which are the least active transporters. As a result, the activation of transmembrane transport becomes a two-step process, which we set out to demonstrate by performing *in situ* irradiation studies in the osmotic assay. For example, when (*E,E,E*)-**1** was postadded to a vesicle solution, initially no significant transport activity was observed due to the poor incorporation into the membrane (Figures 6A and S52). Irradiation with 385 nm light did not immediately lead to an appreciable increase in activity; however, when irradiation was halted, the transport process started. This is explained by the fact that, while promoting membrane incorporation, UV irradiation produces the less mobile (*Z*_PSS_)-form and therefore, not until it isomerizes back to (*E,E,E*)-**1** in the dark, significant chloride efflux is observed. During irradiation, the transport process is thus suppressed, either at the beginning of the run or in a later stage, by exposing the solution to the same wavelength that was initially used to promote membrane incorporation. Using the same light source, the transport process can thus be initiated (to trigger membrane incorporation) as well as deactivated (to reduce the transport activity), which is a unique feature.

In addition, to achieve higher spatiotemporal precision, it would be beneficial to activate transport with two separate wavelengths, which can be achieved with transporter **2**. As this compound does not rapidly isomerize back to the (*E,E,E*)-isomer in the dark, visible light (455 nm) irradiation is needed to generate the more mobile (*E,E,E*)-isomer and activate transport, after the UV irradiation step that promotes incorporation (Figures 6B and S53). These *in situ* experiments nicely illustrate control of transport by altering both membrane incorporation ability and mobility, using one or two irradiation wavelengths.

## Conclusions

In conclusion, functionalization of tren-based tripodal tris-thiourea with azobenzene photoswitches enables the dynamic modulation of transport activity. We showed that light- and thermally induced isomerization causes only a minimal change in chloride binding affinity, while membrane mobility and incorporation are majorly affected. That is, the (*E*,*E*,*E*)-isomers poorly incorporate into the bilayer when postadded (from organic solvent) to the vesicle solution, while the (*Z*_PSS_)-isomers incorporate well. Yet, when preincorporated, the (*E*,*E*,*E*)-isomers turned out to be more active in transport than the respective (*Z*_PSS_)-isomers owing to a higher mobility. We demonstrated that these opposite effects of photoisomerization on transport capability offer unique possibilities to activate and deactivate transport *in situ*, either by using single- or dual-wavelength irradiation, depending on the thermal stability of the azobenzene motif. Importantly, this work demonstrates the first example of dynamic control over the mobility of a transporter,^21^ instead of control over the usually targeted binding affinity. Our results will prove important in the future design and development of photoswitchable transport systems. We envision that mobility control will allow, for example, local (im)mobilization of synthetic transporters in membranes, with consequences for their biological functioning. In addition, it may enable directed motion and active transport, which will be the subject of future studies in our laboratory.

## Data Availability

The data that support the findings of this study are available within the manuscript and its Supporting Information. Crystallographic data can be obtained free of charge from https://www.ccdc.cam.ac.uk/ under CCDC deposition numbers.

## Supporting Information Available

Experimental procedures for the synthesis of **1** and **2** and full characterization, ^1^H NMR and UV–vis studies, transport assays, and X-ray analysis (CCDC 2371925) can be found in the Supporting Information (PDF)
