# Supplementary material for: Activity Control of a Synthetic Transporter by Photodynamic Modulation of Membrane Mobility and Incorporation
Source: J Am Chem Soc. 2024 Nov 1;146(45):31085–93. doi: 10.1021/jacs.4c10952 (PMC11565646; doi:10.1021/jacs.4c10952)
Supplement: Supplementary file 1 — ja4c10952_si_001.pdf [file ja4c10952_si_001.pdf]

Supporting Information

Activity Control of a Synthetic Transporter by Photodynamic Modulation of Membrane Mobility and Incorporation

Jasper E. Bos,[a] Maxime A. Siegler,[b] and Sander J. Wezenberg[a]*

[a] *Leiden Institute of Chemistry, Leiden University, Einsteinweg 55, 2333 CC Leiden, The Netherlands*

[b] *Department of Chemistry, Johns Hopkins University, 3400 N. Charles St., Baltimore, MD, 21218, United States*

Email: s.j.wezenberg@lic.leidenuniv.nl

Table of Contents

Experimental section S2

^{1}H and ^{13}C NMR spectra of new compounds S5

UV-Vis photoisomerization studies in solution S9

UV-Vis photoisomerization studies in vesicles S12

^{1}H NMR photoisomerization studies S16

^{1}H NMR titrations and data fitting S20

HPLC separation of isomers S26

Transmembrane transport experiments S28

Vesicle preparation – HPTS assay S28

Hill analysis – HPTS assay S31

In situ irradiation – HPTS assay S33

Vesicle preparation – Osmotic assay S34

Hill analysis – Osmotic assay S41

In situ irradiation – Osmotic assay S46

Single crystal X-ray crystallography S48

References S51

Experimental section

General methods and materials: CH_2Cl_2 was dried using an Innovative Technology solvent purification systems. DMSO-d_6 and MeCN-d_3 were purchased from Eurisotop. Tris-(2-isothiocyanatoethyl)amine **S1**[1] and 4-aminomethylazobenzene **S3**[2] were prepared according to procedures described in the literature. All other chemicals were commercial products and were used as received. Thin-layer chromatography (TLC) was carried out on aluminum sheets coated with silica 60 F254. Compounds were visualized with UV light (254 nm) or by staining with ninhydrin. Melting points (m.p.) were determined using a Büchi-M560 melting point apparatus. ^{1}H, and ^{13}C spectra were recorded on Bruker AV 400, Bruker 500 Ultra Shield and Bruker AV 600 instruments at 294 K unless indicated otherwise. Chemical shifts (δ) are denoted in parts per million (ppm) relative to residual protiated solvent (DMSO-d_6: for ^{1}H detection, $\delta = 2.50$ ppm; for ^{13}C detection, $\delta = 39.52$ ppm). The splitting pattern of peaks is designated as follows: s (singlet), d (doublet), t (triplet), m (multiplet), and br (broad). High-resolution mass spectrometry (ESI-MS) was performed on a Thermo Scientific Q Exactive HF spectrometer with ESI ionization. IR spectra were recorded on a Perkin Elmer Spectrum Two FT-IR spectrometer. The wavenumber (v) is in units of reciprocal centimeters (cm^{-1}) and the intensity ($v = cm^{-1}$) is designated as follows: s (strong), m (medium), w (weak), very w (very weak), br (broad), and sh (shoulder). UV-Vis spectra were recorded on an Agilent Cary 8454 spectrometer using 1 cm quartz cuvettes. Fluorescence was measured on a JASCO FP-8500 spectrofluorimeter using 1 cm PS cuvettes. HPLC analysis was performed using a Shimadzu Prominence-i LC-2030C 3DPlus system equipped with a PDA detector and a Phenomenex Gemini C18 column, 4.6 × 50 mm, 3 μm particle size. Irradiation of UV-Vis and NMR samples was carried out using Thorlabs model M365F1 (4.1 mW), M385F1 (10.7 mW), M455F3 (24.5 mW) LEDs positioned at a distance of 1 cm to the sample. Fluorescence samples were irradiated from the top by mounting the LEDs on the fluorimeter lid.[3]

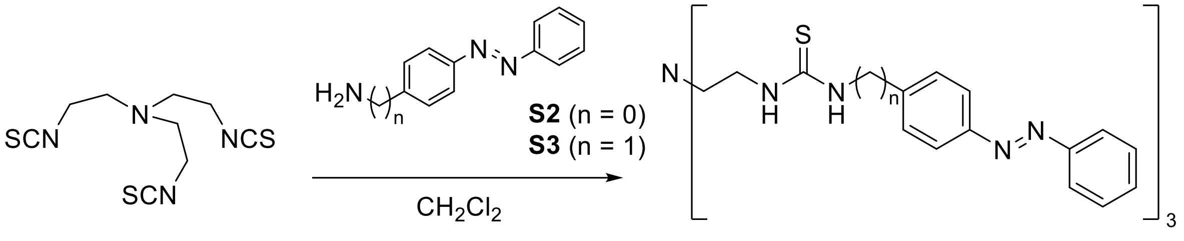

S1

(*E,E,E*)-**1**: n = 0 (77%)
(*E,E,E*)-**2**: n = 1 (73%)

Scheme S1: Synthesis of azobenzene-appended tren-based tris-thioureas (*E,E,E*)-**1** and (*E,E,E*)-**2**

1,1',1"-[Nitrilotris(ethane-2,1-diyl)]tris[3-(4-{(*E*)-phenyldiazenyl}phenyl)thiourea] [(*E,E,E*)-1]

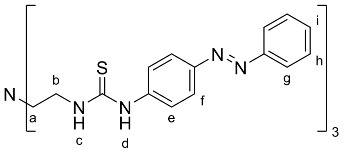

Tris-(2-isothiocyanatoethyl)amine (110 mg, 0.40 mmol) and 4-aminoazobenzene (233 mg, 1.18 mmol) were dissolved in anhydrous CH_2Cl_2 (10 mL) and the solution was kept at reflux for 7 days (with occasional addition of CH_2Cl_2) under a N_2 atmosphere. The volatiles were removed in vacuo and the product was purified by column chromatography (SiO_2, CH_2Cl_2/MeOH gradient 99:1 to 95:5), followed by recrystallization from $CHCl_3$/MeOH to afford (*E,E,E*)-**1** (260 mg, 77 %) as orange crystals; R_f = 0.32 (SiO_2, CH_2Cl_2/MeOH 95:5); m.p. 205.8 °C; ^{1}H NMR (500 MHz, DMSO-d_6, assignment based on 2D COSY and NOESY): δ 9.98 (s, 3H; H**d**), 7.98 (br. s, 3H; H**c**), 7.84 (m, 12H; H**f** and H**g**), 7.71 (d, J = 8.8 Hz; 6H, H**e**), 7.58 – 7.51 (m; 9H, H**h** and H**i**), 3.68 (br. s, 6H; H**b**), 2.83 (t, J = 6.8 Hz; 6H, H**a**) ppm; ^{13}C{^{1}H} NMR (126 MHz, DMSO-d_6, APT) δ 180.0, 152.0, 147. 7, 142.7, 131.1, 129.4, 123.4, 122.3, 122.0, 51.9, 41.9 ppm; IR (ATR): ν = 3331 (very w), 3270 (w, sh), 3225 (br. w), 3135 (very w), 3098 (very w), 3065 (very w), 3025 (very w), 2960 (very w), 2941 (very w), 2925 (very w), 2886 (very w), 2809 (very w), 2742 (very w), 1602 (w), 1591 (w), 1554 (s), 1536 (s), 1522 (s), 1496 (s), 1466 (w), 1454 (w), 1445 (w), 1416 (w), 1389 (w), 1370 (w), 1330 (m), 1308 (s), 1297 (s), 1269 (m), 1241 (s), 1219 (m, sh), 1195 (w, sh), 1151 (s), 1139 (m), 1118 (m), 1105 (w), 1091 (m), 1070 (w), 1051 (w), 1044 (w) 1019 (w), 1004 (m), 991 (w, sh), 963 (very w), 944 (very w), 929 (very w), 918 (very w), 900 (very w), 884 (very w), 859 (very w, sh), 842 (m), 831 (w, sh), 821 (very w), 806 (very w), 768 (m), 737 (very w), 722 (very w), 711 (very w), 696 (m, sh), 689 (s), 664 (m, sh), 652 (m), 639 (w), 623 cm^{-1} (w); HRMS (ESI) *m/z*: 864.3151 ($[M+H]^+$ calcd for $C_{45}H_{46}N_{13}S_3^+$: 864.3156)

1,1',1"-[Nitrilotris(ethane-2,1-diyl)]tris[3-(4-{[*E*]-phenyldiazenyl}benzyl)thiourea] [(*E*,*E*,*E*)-2]

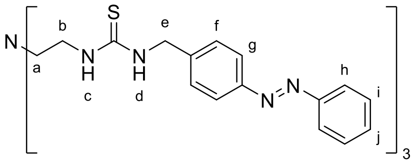

Tris-(2-isothiocyanatoethyl)amine (54 mg, 0.20 mmol), 4-aminomethylazobenzene (152 mg, 0.72 mmol) and triethylamine (100 µL, 0.73 mmol) were dissolved in anhydrous CH_2Cl_2 (1.5 mL) and the solution was heated to 70°C for 72 h in a pressure tube under a N_2 atmosphere. The volatiles were removed in vacuo and the purified by reversed-phase column chromatography (C_{18}-functionalized silica, H_2O/MeCN gradient 70:30 to 8:92) to afford (*E*,*E*,*E*)-**2** (131 mg, 73%) as an orange solid. Analytically pure samples were obtained by recrystallisation from MeCN; R_f = 0.24 (SiO_2, CH_2Cl_2/MeOH 95:5); m.p. 175.7 °C; ^{1}H NMR (600 MHz, DMSO-d_6, assignment based on 2D COSY and NOESY): δ 8.07 (br. s, 3H; H**d**), 7.87 – 7.84 (m, 12H; H**g** and H**h**), 7.60 – 7.54 (m, 9H; H**i** and H**j**), 7.54 – 7.45 (d, J = 8.1 Hz; 9H, H**c** and H**f**), 4.77 (br. s, 6H; H**e**), 3.55 (br. s, 6H; H**b**), 2.69 (t, J = 6.8 Hz, 6H; H**a**) ppm; ^{13}C{^{1}H} NMR (151 MHz, DMSO-d_6) δ 182.8, 151.9, 150.9, 143.4, 131.4, 129.5, 128.1, 122.6, 122.5, 52.5 46.6, 41.9 ppm; IR (ATR): ν = 3323 (very w), 3259 (br. w), 3198 (very w, sh), 3095 (very w), 3049 (very w), 3030 (very w), 2979 (very w), 2920 (very w), 2901 (very, w), 2881 (very w), 2829 (very w, sh), 2813 (very w), 1603 (very w), 1581 (very w), 1543 (s), 1525 (s, sh), 1500 (m), 1485 (w), 1476 (w), 1444 (w), 1415 (m), 1380 (w, sh), 1367 (m), 1341 (m), 1314 (w), 1287 (s), 1279 (s), 1267 (m), 1250 (m), 1221 (m), 1197 (w), 1176 (w), 1151 (w), 1144 (w), 1124 (m), 1101 (w), 1071 (m), 1048 (w), 1036 (w), 1018 (w, sh), 1012 (w), 998 (very w), 967 (w), 942 (m), 923 (very w), 907 (very w), 879 (very w), 822 (m), 779 (very w), 763 (m), 705 (w, sh), 685 (s), 654 (m), 612 cm^{-1} (w); HRMS (ESI) *m/z*: 906.36253 ([M+H]$^+$ calcd for $C_{48}H_{52}N_{13}S_3^+$: 906.3625)

^{1}H and ^{13}C NMR spectra of new compounds

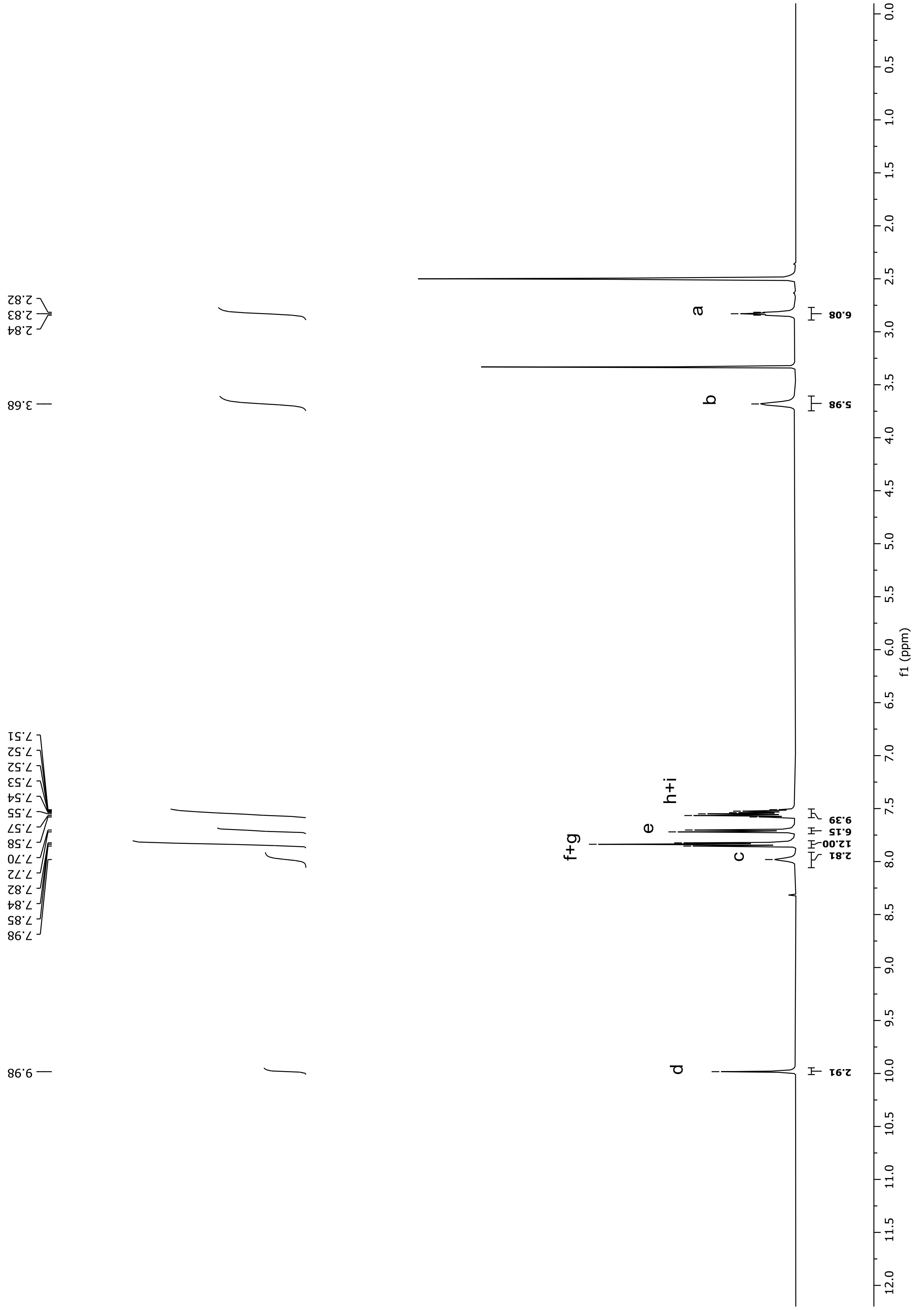

Figure S1. 500 MHz ^{1}H NMR spectrum of (*E*,*E*,*E*)-**1** in DMSO-d_6

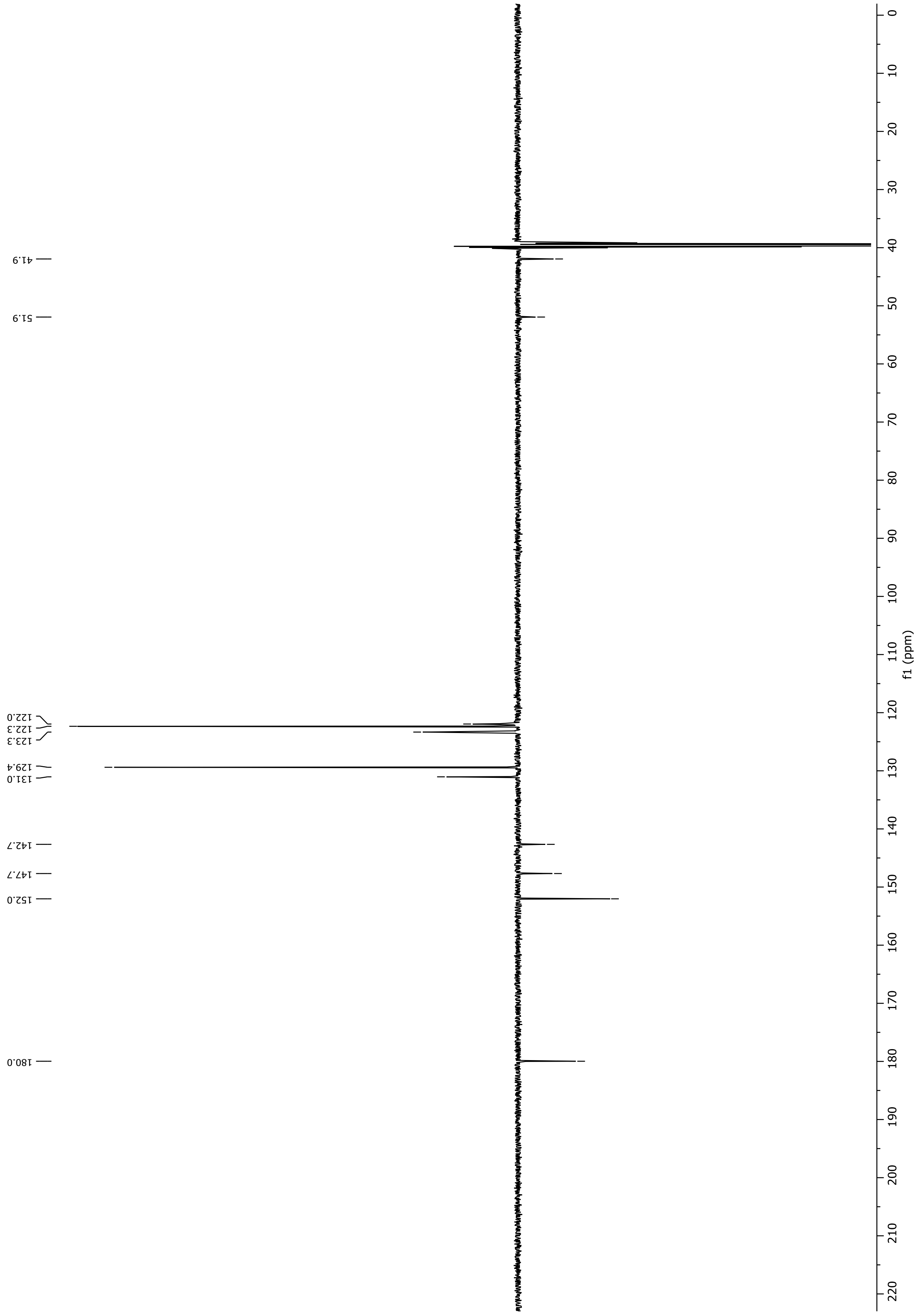

Figure S2. 126 MHz $^{13}C\{^{1}H\}$ NMR (APT) spectrum of (*E*,*E*,*E*)-**1** in DMSO-d_6

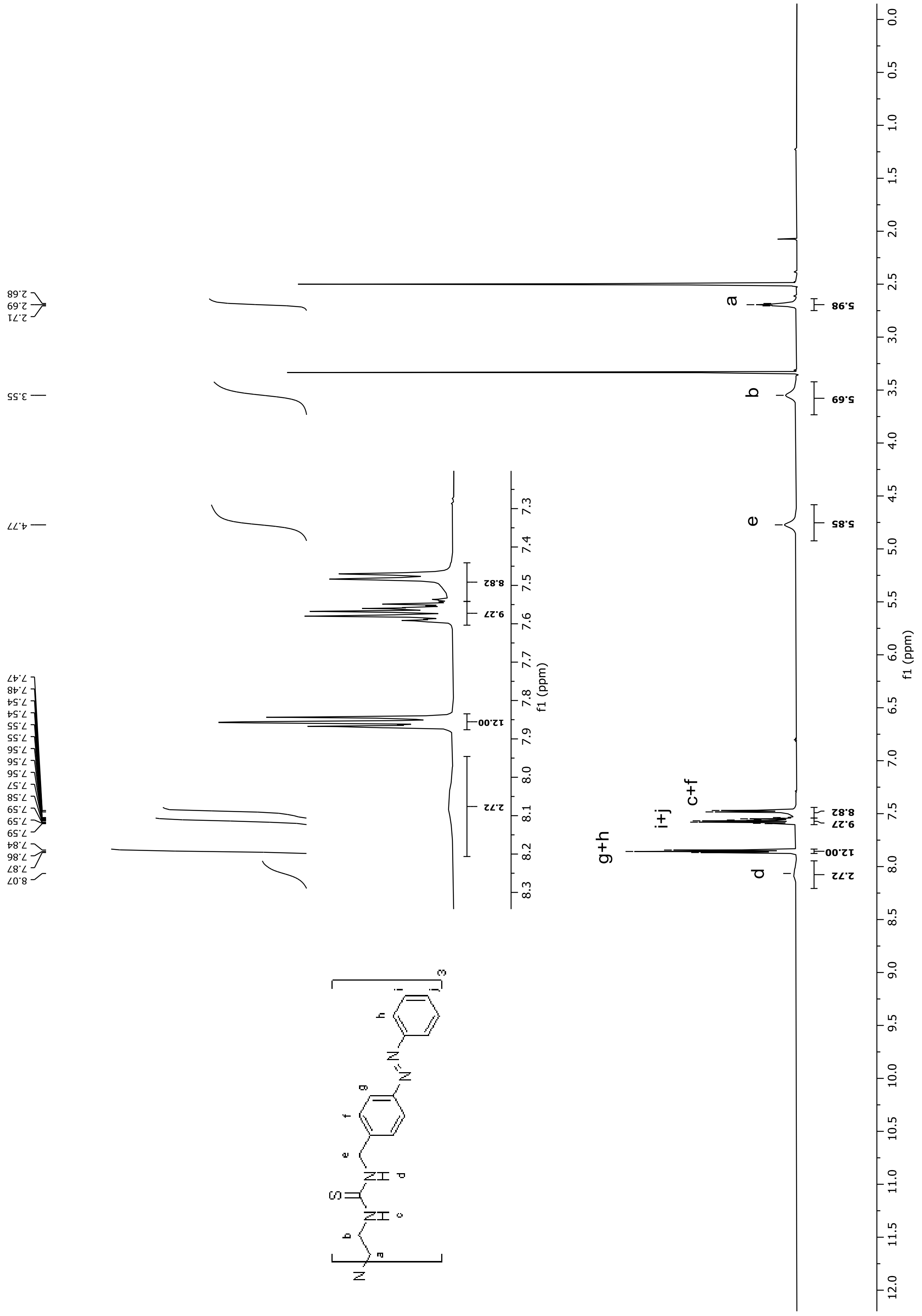

Figure S3. 600 MHz ^{1}H NMR spectrum of (E,E,E)-**2** in DMSO-d_6

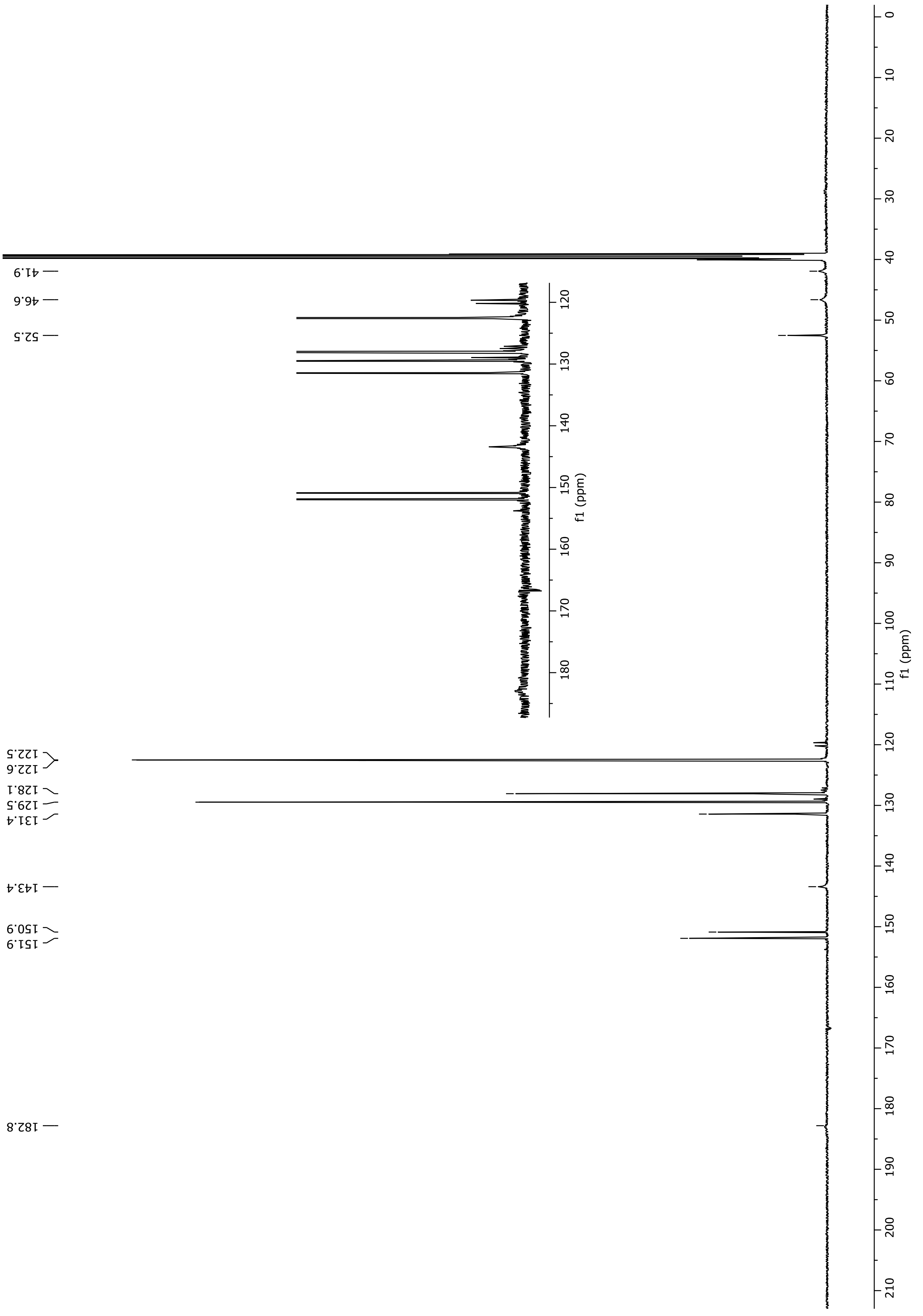

Figure S4. 151 MHz $^{13}C\{^{1}H\}$ NMR spectrum of (*E*,*E*,*E*)-**2** in DMSO-d_6

UV-Vis photoisomerization studies in solution

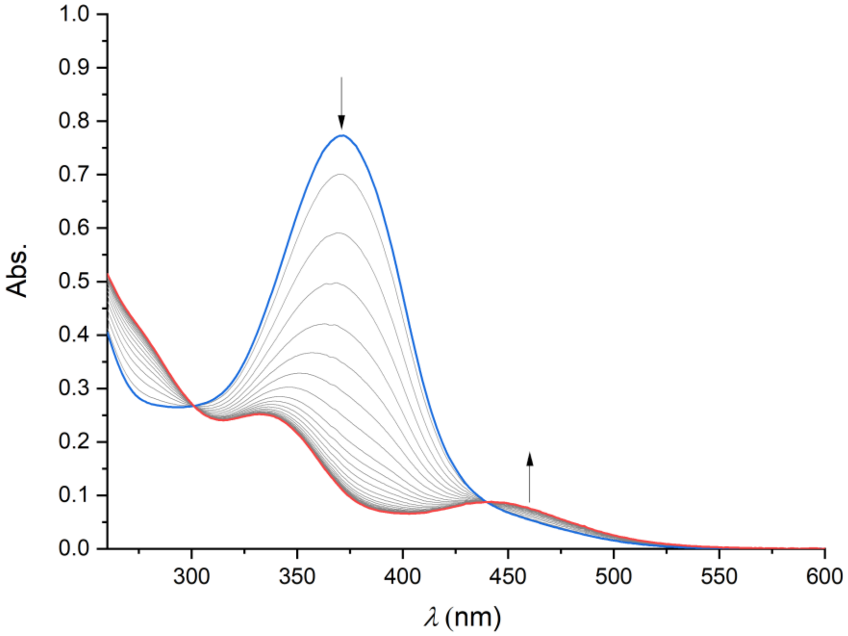

Figure S5. UV-Vis spectral changes of (*E*,*E*,*E*)-**1** (1.0×10^{-5} M, 1 cm quartz cuvette) in DMSO/MeCN (1:1 *v*/*v*) upon irradiation with 385 nm light. Recorded at 0.5 s intervals.

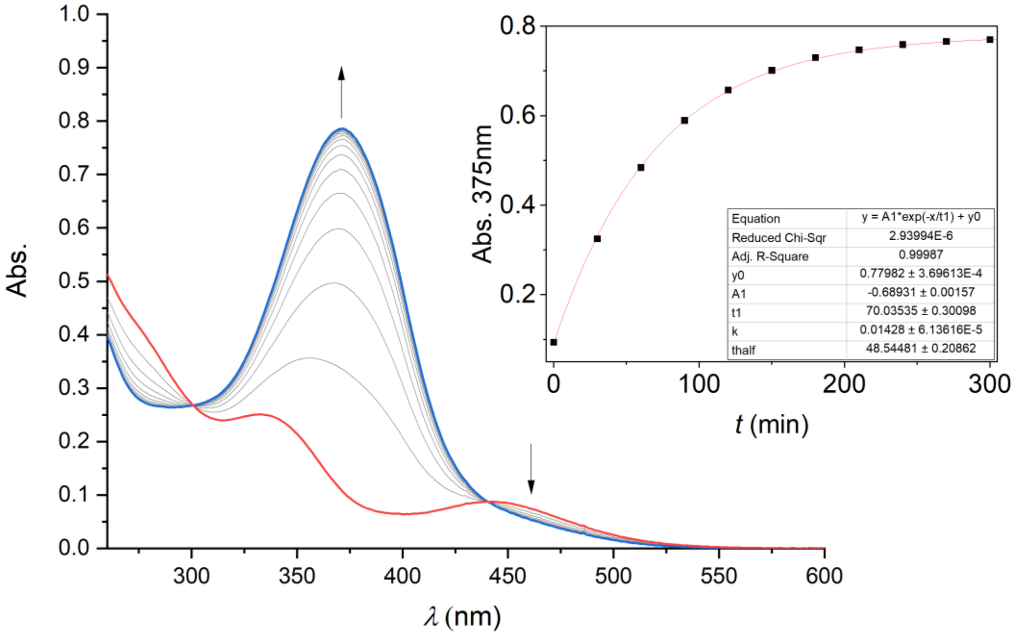

Figure S6. UV-Vis spectral changes of (Z_{PSS})-**1** (1.0×10^{-5} M, 1 cm quartz cuvette) in DMSO/MeCN (1:1 *v*/*v*) when kept in the dark. Recorded at 30 min intervals. The inset shows the change in absorption (λ = 375 nm) over time, fitted to a first order exponential decay function ($A = A_0e^{-t/t1} + y0$); $k = 0.0143$ min^{-1}.

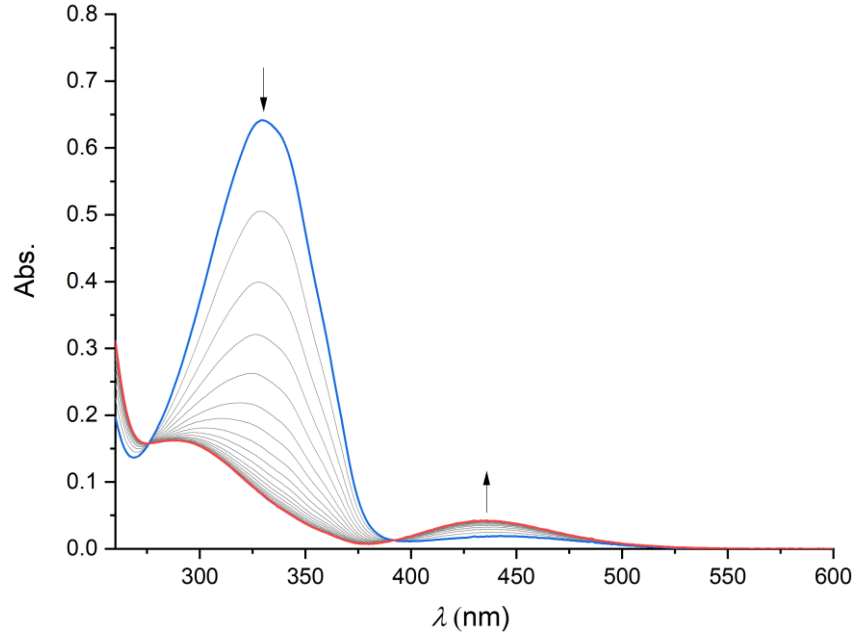

Figure S7. UV-Vis spectral changes of (*E*,*E*,*E*)-**2** (1.0×10^{-5} M, 1 cm quartz cuvette) in DMSO/MeCN (1:1 *v*/*v*) upon irradiation with 365 nm light. Recorded at 3 s intervals.

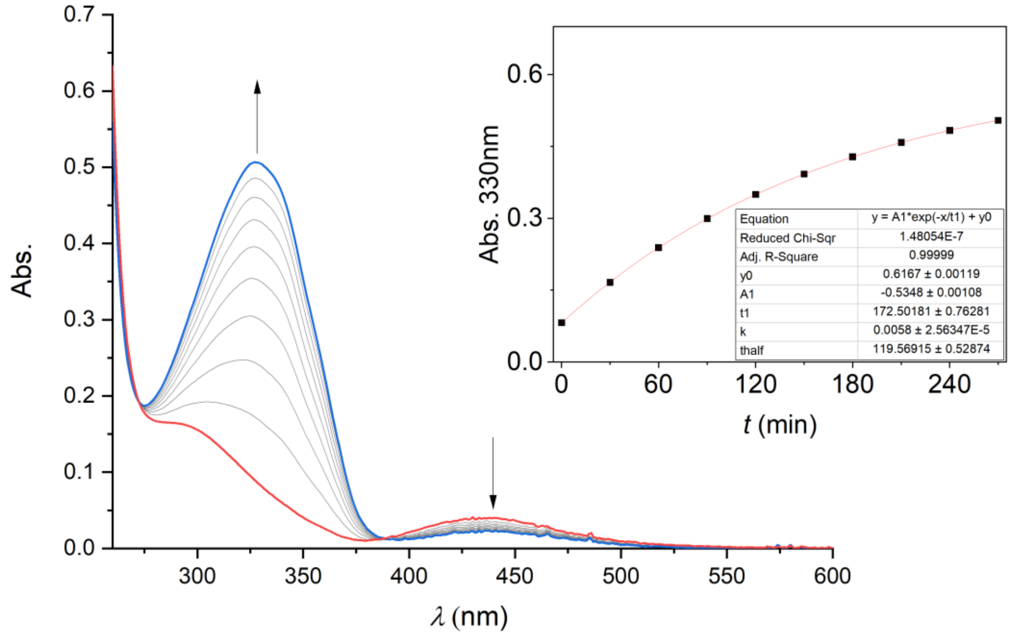

Figure S8. UV-Vis spectral changes of (Z_{PSS})-**2** (1.0×10^{-5} M, 1 cm quartz cuvette) in DMSO/MeCN (1:1 *v*/*v*) when kept in the dark at 333 K. Recorded at 30 min intervals. The inset shows the change in absorption (λ = 330 nm) over time, fitted to a first order exponential decay function ($A = A_0e^{-t/t1} + y0$); k = 0.0058 min^{-1}.

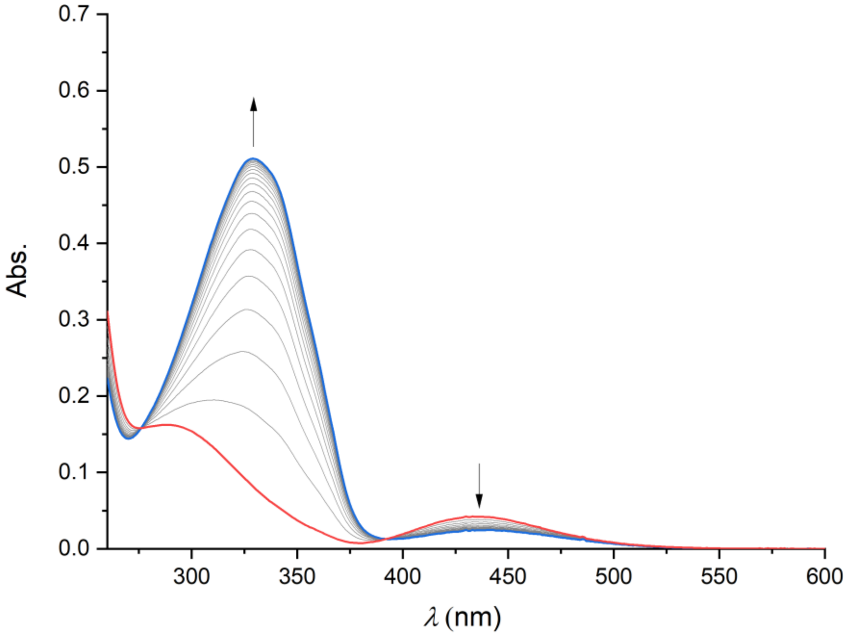

Figure S9. UV-Vis spectral changes of (Z_{PSS})-**2** (1.0 × 10^{-5} M, 1 cm quartz cuvette) in DMSO/MeCN (1:1 *v*/*v*) upon irradiation with 455 nm light. Recorded at 3 s intervals.

UV-Vis photoisomerization studies in vesicles

The transporters were pre-incorporated at 1.0 mol% (transporter to lipid) according to the general procedure for the osmotic assay described on page S32, with the modification that extrusion was done with a 200 nm membrane. Vesicle stock solutions were obtained with a lipid concentration of 2.6 – 3.0 mM. For the measurements the stock solution of vesicles containing KCl (300 mM) were suspended in KGlu (300 mM) (with all solutions buffered to pH 7.2 with HEPES (10 mM)) to a standard volume (2.2 mL) in a quartz cuvette, obtaining a solution with a concentration of 0.4 mM in lipids. Note that the scattering of light by the liposomes gives an artifact at the irradiation wavelength.

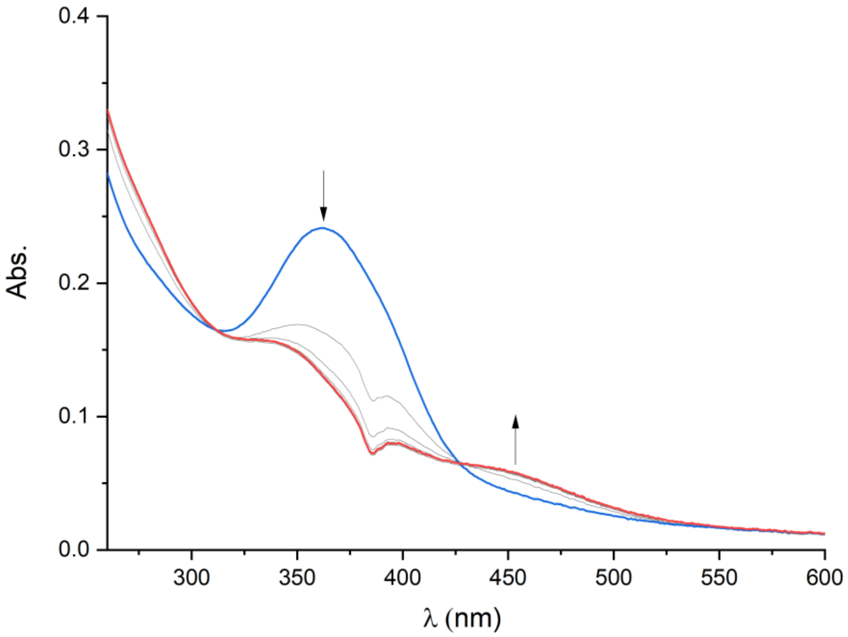

Figure S10. UV-Vis spectral changes of (*E*,*E*,*E*)-**1** (4.0×10^{-6} M, 1 cm quartz cuvette) incorporated at 1.0 mol% in liposomes assembled from POPC lipids (4.0×10^{-4} M) upon irradiation with 385 nm light. Recorded at 3 s intervals.

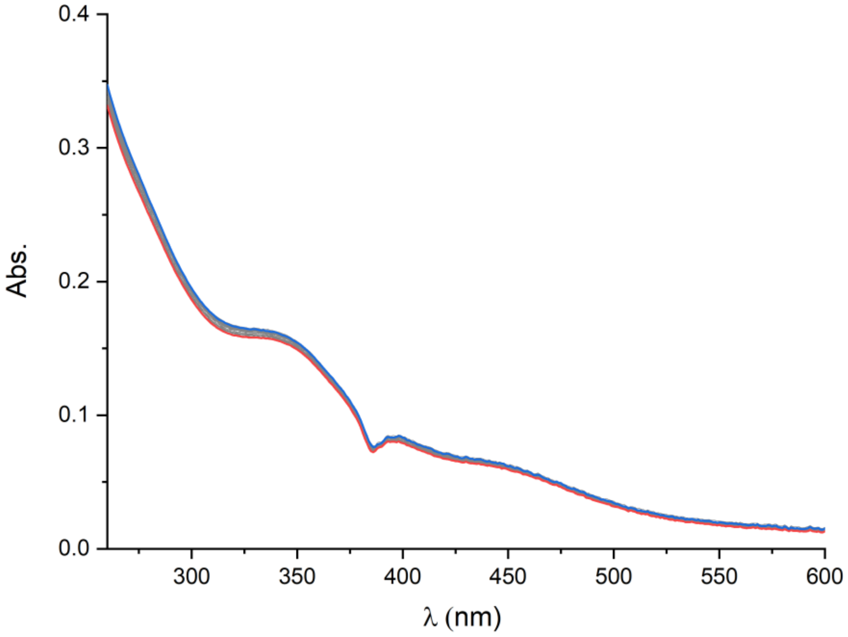

Figure S11. UV-Vis spectra of (Z_{PSS})-**1** (4.0×10^{-6} M, 1 cm quartz cuvette) incorporated at 1.0 mol% in liposomes assembled from POPC lipids (4.0×10^{-4} M) upon continued irradiation with 385 nm light for 15 minutes recorded at a 30 s interval, confirming their integrity.

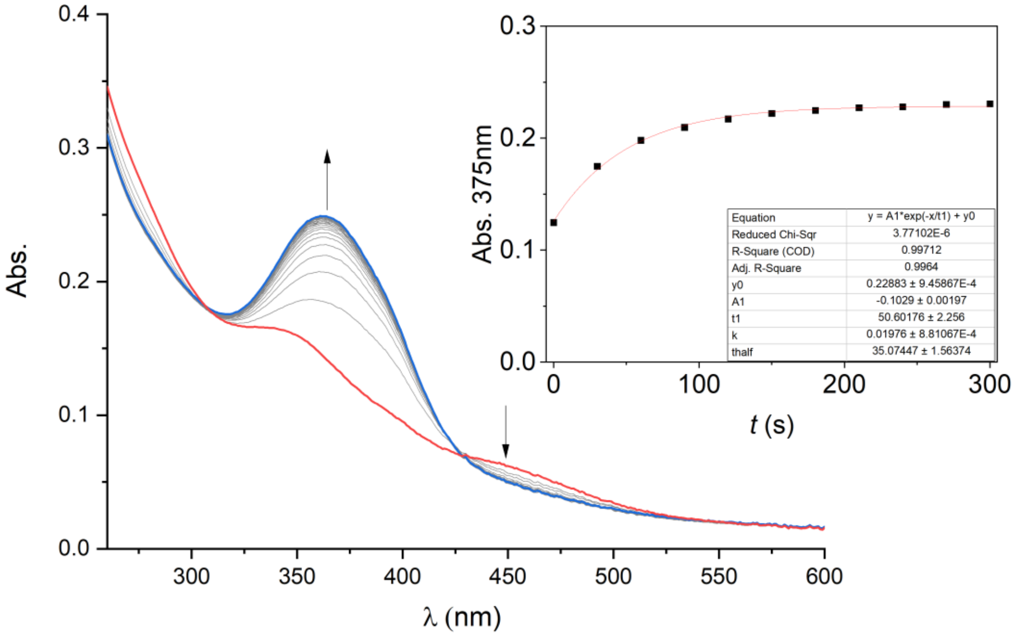

Figure S12. UV-Vis spectral changes of (Z_{PSS})-**1** (4.0×10^{-6} M, 1 cm quartz cuvette) incorporated at 1.0 mol% in liposomes assembled from POPC lipids (4.0×10^{-4} M) when kept in the dark. The inset shows the change in absorption (λ = 375 nm) over time, fitted to a first order exponential decay function ($A = A_0e^{-t/t1} + y0$); $k = 0.0198\ s^{-1}$.

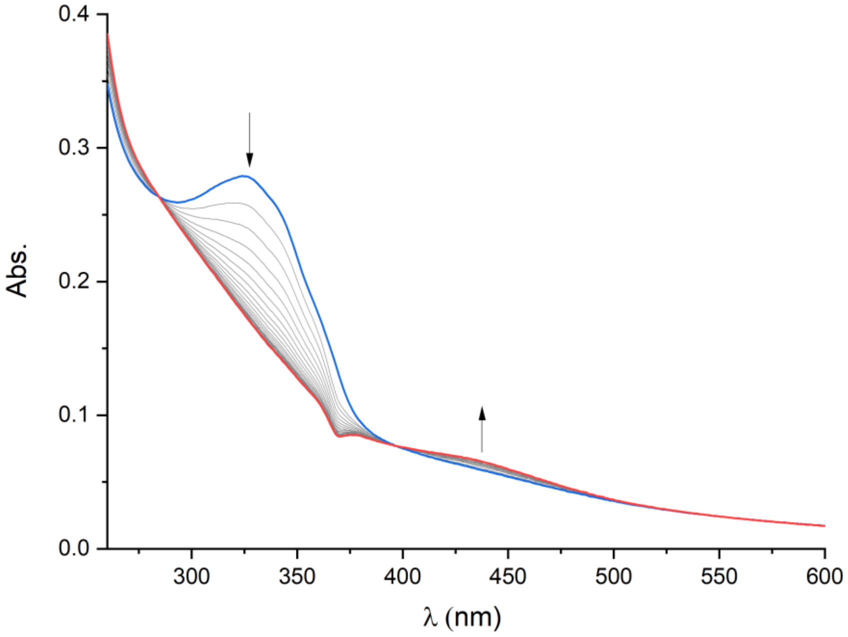

Figure S13. UV-Vis spectral changes of (*E*,*E*,*E*)-**2** (4.0×10^{-6} M, 1 cm quartz cuvette) incorporated at 1.0 mol% in liposomes assembled from POPC lipids (4.0×10^{-4} M) upon irradiation with 365 nm light. Recorded at 3 s intervals.

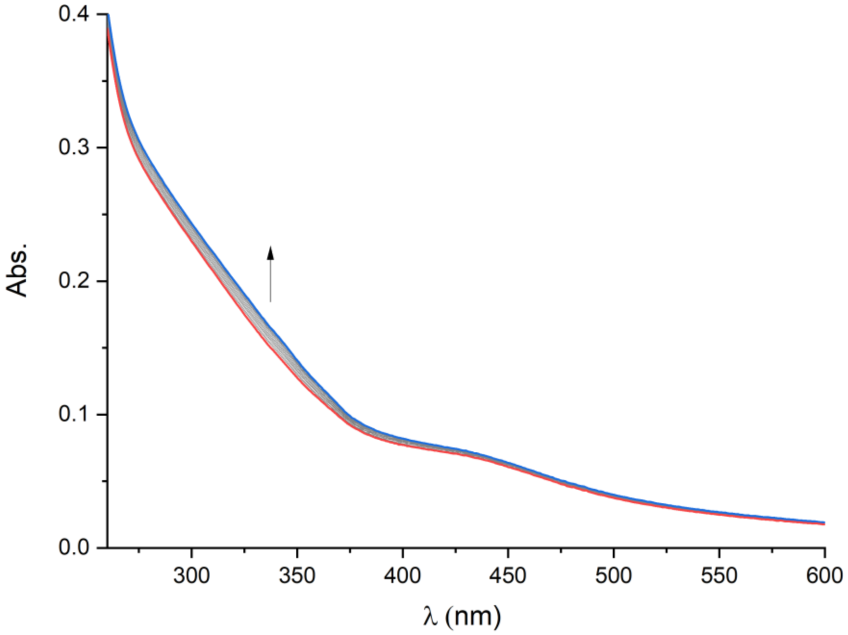

Figure S14. UV-Vis spectral changes of (Z_{PSS})-**2** (4.0×10^{-6} M, 1 cm quartz cuvette) incorporated at 1.0 mol% in liposomes assembled from POPC lipids (4.0×10^{-4} M) when kept in the dark for 1 hour. Recorded at 5 min intervals. The experiment shows that minimal thermal isomerization occurs during the timeframe used for the osmotic assay.

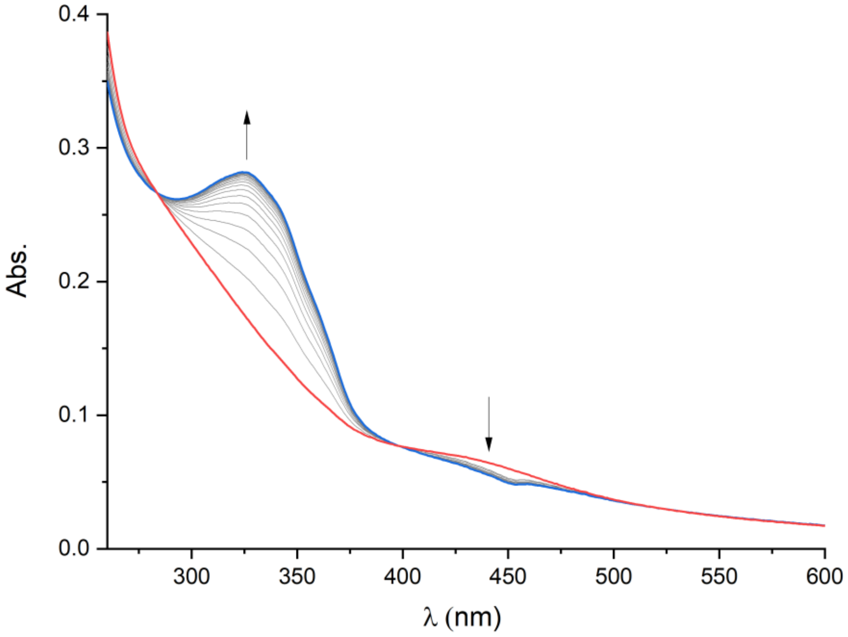

Figure S15. UV-Vis spectral changes of (Z_{PSS})-**2** (4.0 × 10^{-6} M, 1 cm quartz cuvette) incorporated at 1.0 mol% in liposomes assembled from POPC lipids (4.0 × 10^{-4} M) upon irradiation with 455 nm light. Recorded at 3 s intervals.

^{1}H NMR photoisomerization studies

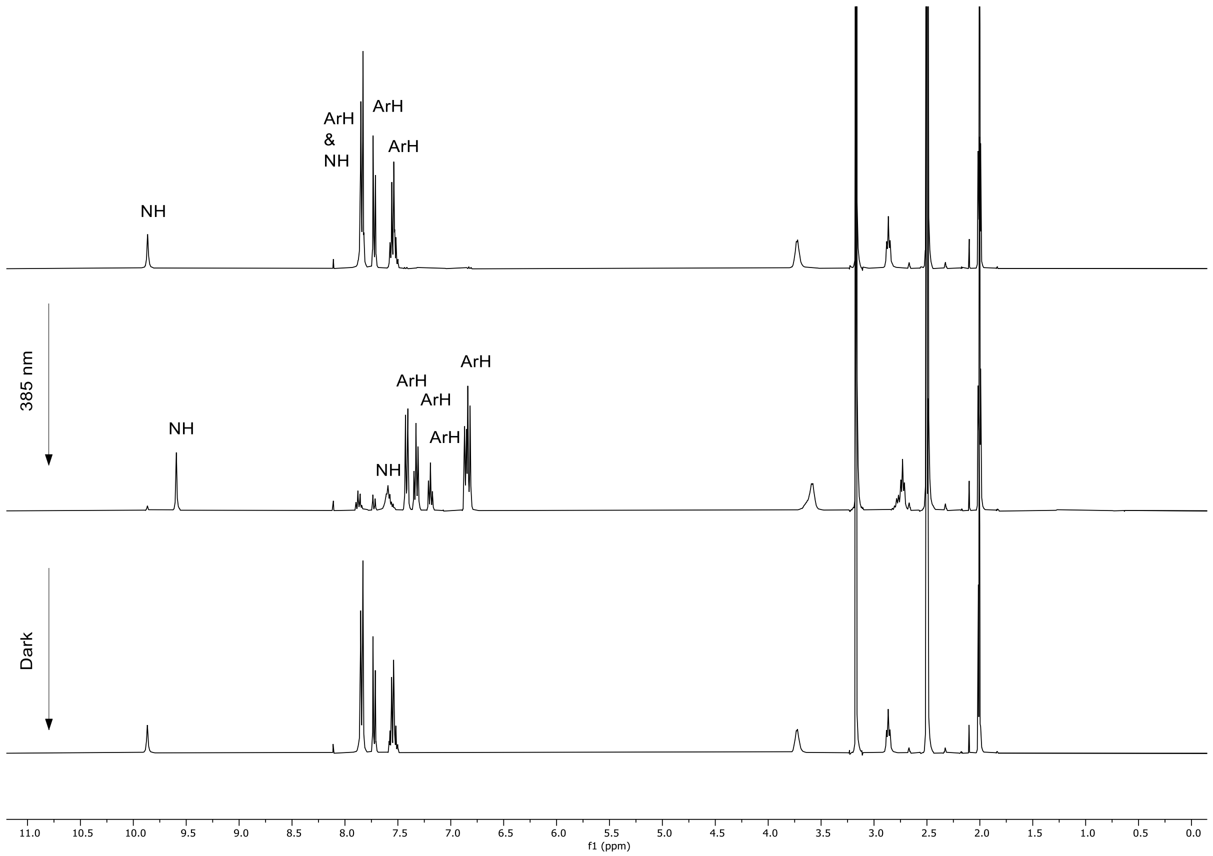

Figure S16. The ^{1}H NMR spectrum (400 MHz, 294 K) of (*E,E,E*)-**1** (2.0 mM DMSO-d_6/MeCN-d_3 1:1 *v*/*v*) before (top) and after irradiation with 385 nm light for 20 min (middle), and after standing in the dark for 24 hours (bottom). The PSS_{385} ratio was calculated by averaging the integrals of the NH and arom. H signals, and determined as 11:89 (*E*/*Z*). The signal at δ = 8.10 belongs to a $CHCl_3$ trace impurity.[4]

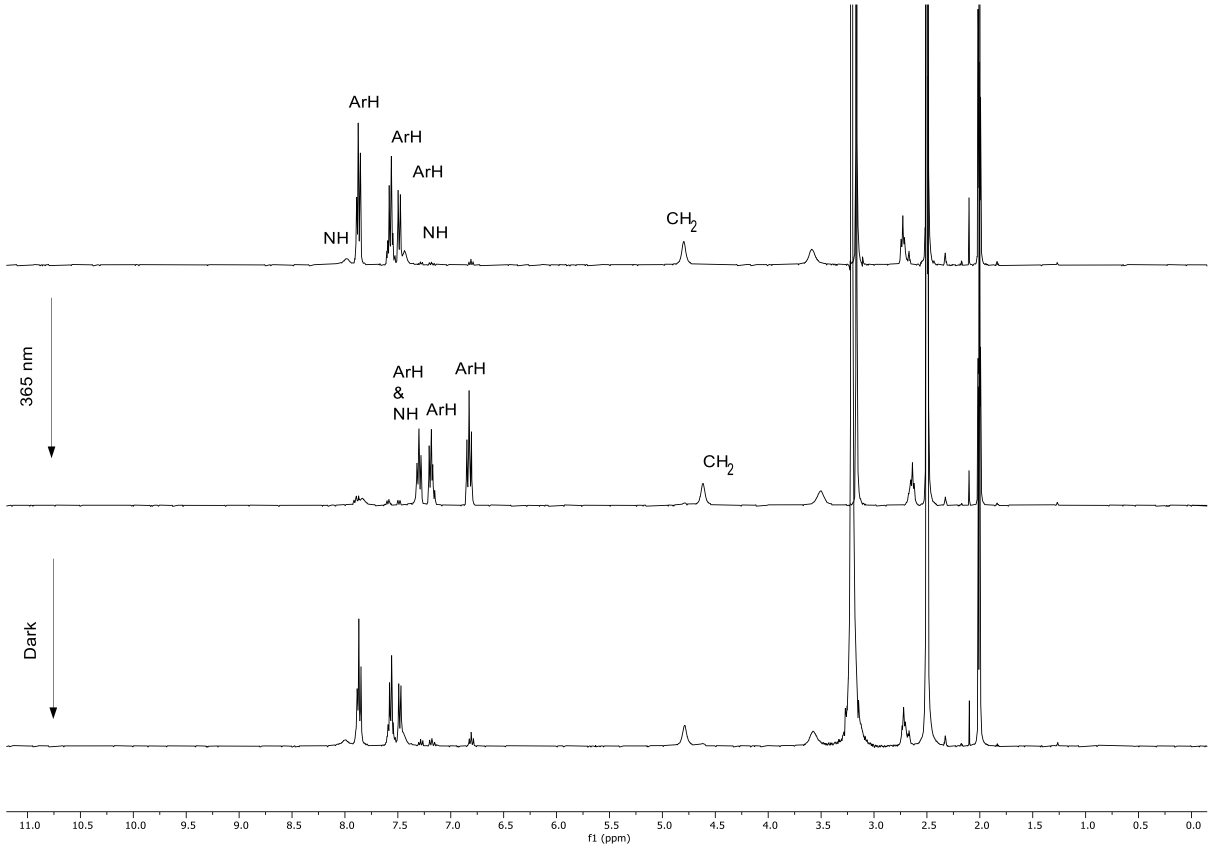

Figure S17. The ^{1}H NMR spectrum (400 MHz, 294 K) of (*E,E,E*)-**2** (2.0 mM DMSO-d_6/MeCN-d_3 1:1 *v*/*v*) before (top) and after irradiation with 365 nm light for 10 min (middle), and after standing in the dark for 61 days (bottom). The PSS_{365} ratio was calculated by averaging the integrals of the NH, CH and arom. H signals, and determined as 7:93 (*E*/*Z*). In the dark the molecule reaches a thermal equilibrium with a ratio of 91:9 (*E*/*Z*).

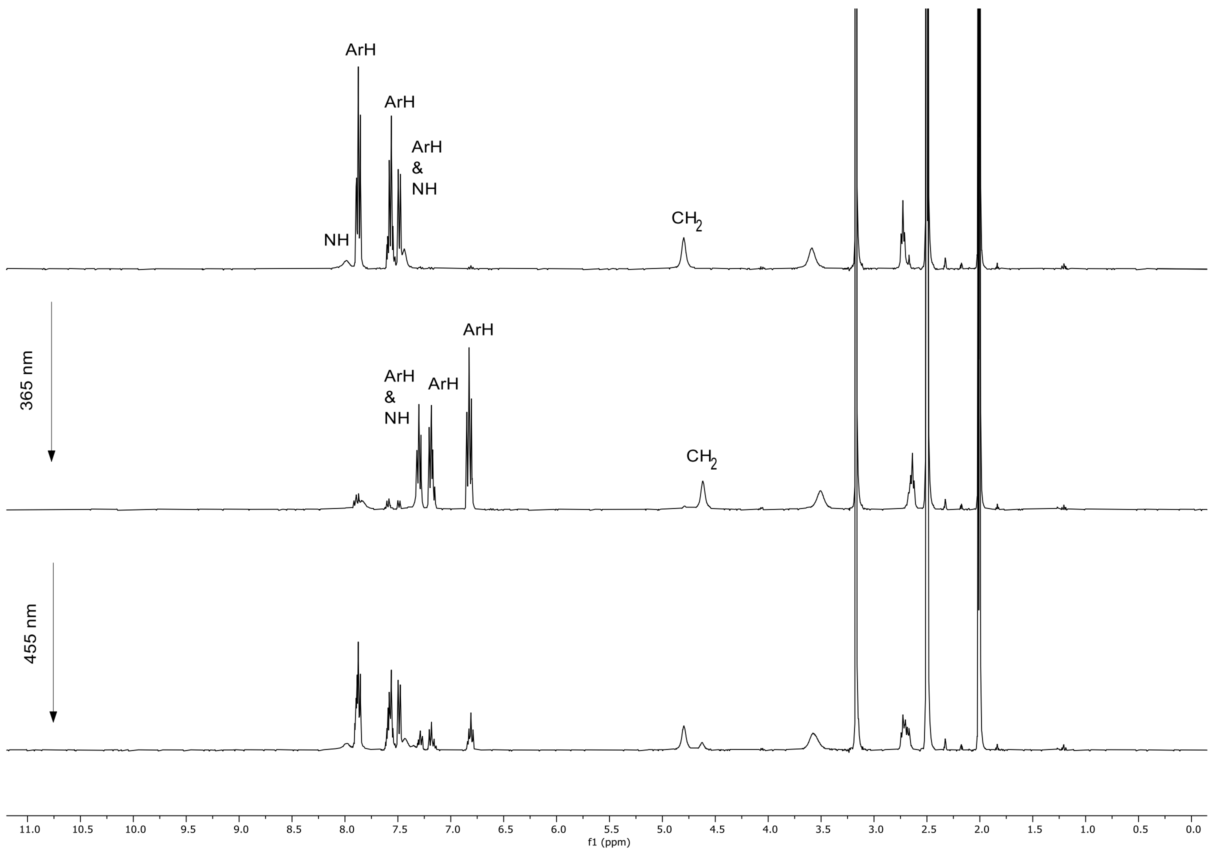

Figure S18. The ^{1}H NMR spectrum (400 MHz, 294 K) of (*E,E,E*)-**2** (2.0 mM DMSO-d_6/MeCN-d_3 1:1 *v*/*v*) before (top) and after irradiation with 365 nm light for 10 min (middle), and after irradiation with 455 nm light for 10 minutes (bottom). The PSS_{455} ratio was calculated by averaging the integrals of the NH, CH and arom. H signals, and determined as 77:23 (*E*/*Z*).

Distribution of isomers

As by ^{1}H NMR spectroscopy the various possible isomers [i.e. (*Z*,*Z*,*Z*), (*Z*,*E*,*E*), (*Z*,*Z*,*E*) and (*E*,*E*,*E*)] could not be distinguished, their relative distribution was calculated based on the overall E/Z ratio, assuming independent switching of the azobenzene moieties.

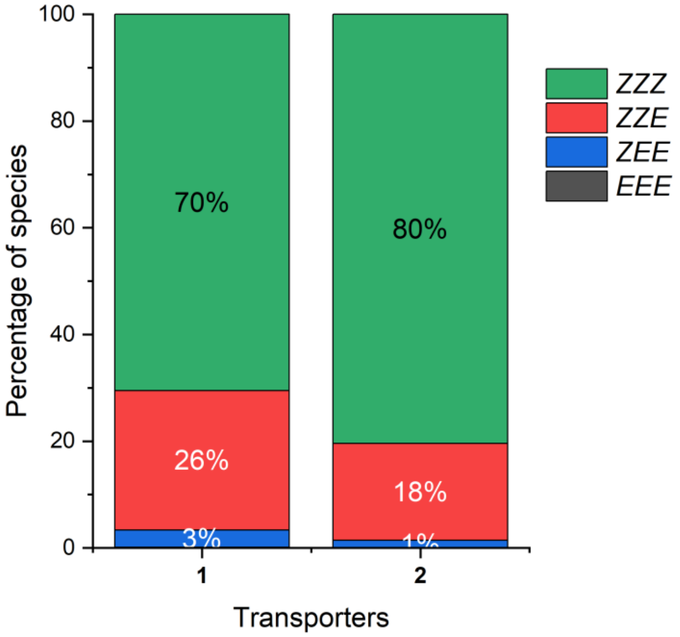

Figure S19. The distribution of isomers at the PSS_{385} and PSS_{365} for transporters **1** and **2**, respectively.

^{1}H NMR titrations and data fitting

First a 5.0 mM solution of the receptor was prepared in DMSO-d_6/0.5%H_2O. In this solution $[Bu_4N]^+[Cl]^-$ was dissolved to get a concentration of approximately 0.1 M. The $[Bu_4N]^+[Cl]^-$ solution was then added stepwise to 0.5 mL of the receptor solution, and after each addition a ^{1}H NMR spectrum (500 MHz) was recorded. To measure binding to (Z_{PSS})-**2** both the receptor and the $[Bu_4N]^+[Cl]^-$ solution were irradiated with 365 nm until PSS was reached.

Since (Z_{PSS})-**2** is a mixture of various isomers (see S19) the observed binding of chloride is the combination of the affinities of the separate isomers present. Since the ^{1}H NMR signals of the isomers cannot be distinguished, it is not possible to obtain all the individual binding constants. Given that the major species are the (*Z*,*Z*,*Z*) and (*Z*,*Z*,*E*) isomers, we included only the NH signals corresponding to *Z* azobenzene in the fit. The apparent binding constant obtained should be interpreted as the average affinity of all *Z* containing isomers present in the PSS mixture. All chemical shift data was fitted to a 1:1 model using HypNMR.[5]

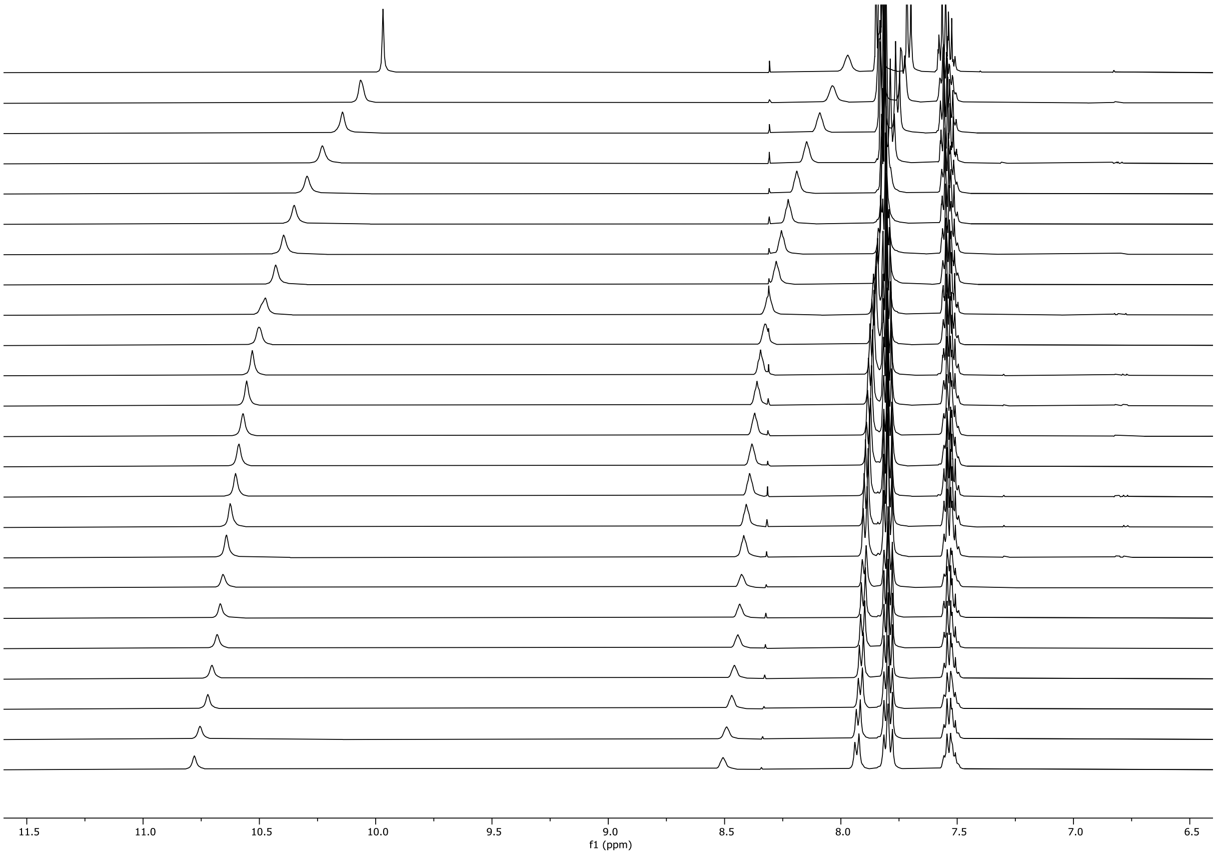

Figure S20. ^{1}H NMR spectral changes (500 MHz, 294 K) in the aromatic region of (*E*,*E*,*E*)-**1** in DMSO-d_6/0.5%H_2O (5.0×10^{-3} M) upon the stepwise addition of $[Bu_4N]^+[Cl]^-$ (from top to bottom: 0.00, 0.21, 0.42, 0.63, 0.83, 1.03, 1.23, 1.42, 1.79, 1.97, 2.32, 2.66, 2.99, 3.30, 3.61, 4.19, 4.74, 5.25, 5.73, 6.19, 7.22, 8.12, 9.62, 10.55 equivalents). The signal at δ = 8.31 belongs to a $CHCl_3$ trace impurity.[4]

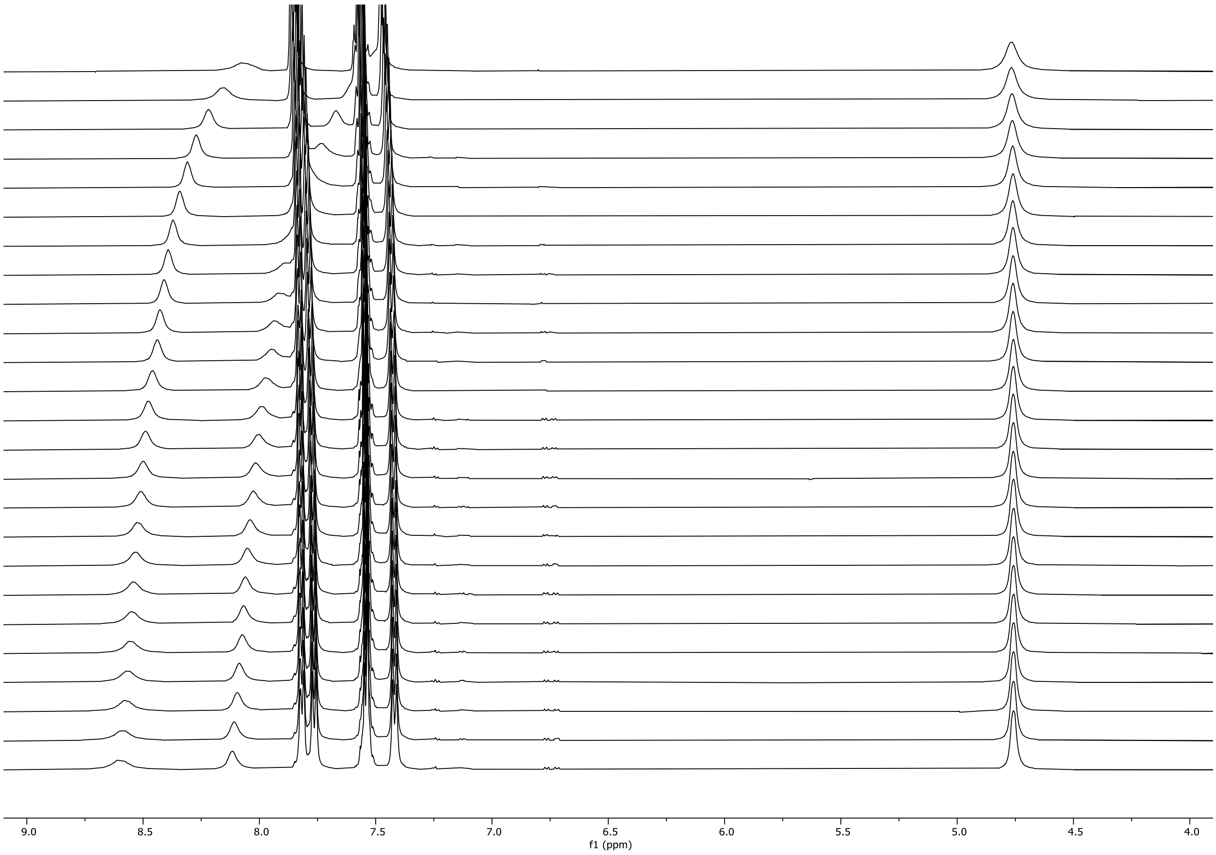

Figure S21. ^{1}H NMR spectral changes (500 MHz, 294 K) in the aromatic region of (*E*,*E*,*E*)-**2** in DMSO-d_6/0.5%H_2O (5.0×10^{-3} M) upon the stepwise addition of $[Bu_4N]^+[Cl]^-$ (from top to bottom: 0.00, 0.24, 0.48, 0.71, 0.94, 1.16, 1.38, 1.60, 1.81, 2.02, 2.22, 2.62, 3.00, 3.37, 3.73, 4.07, 4.73, 5.34, 5.92, 6.47, 6.98, 8.14, 9.16, 10.86, 12.27 equivalents).

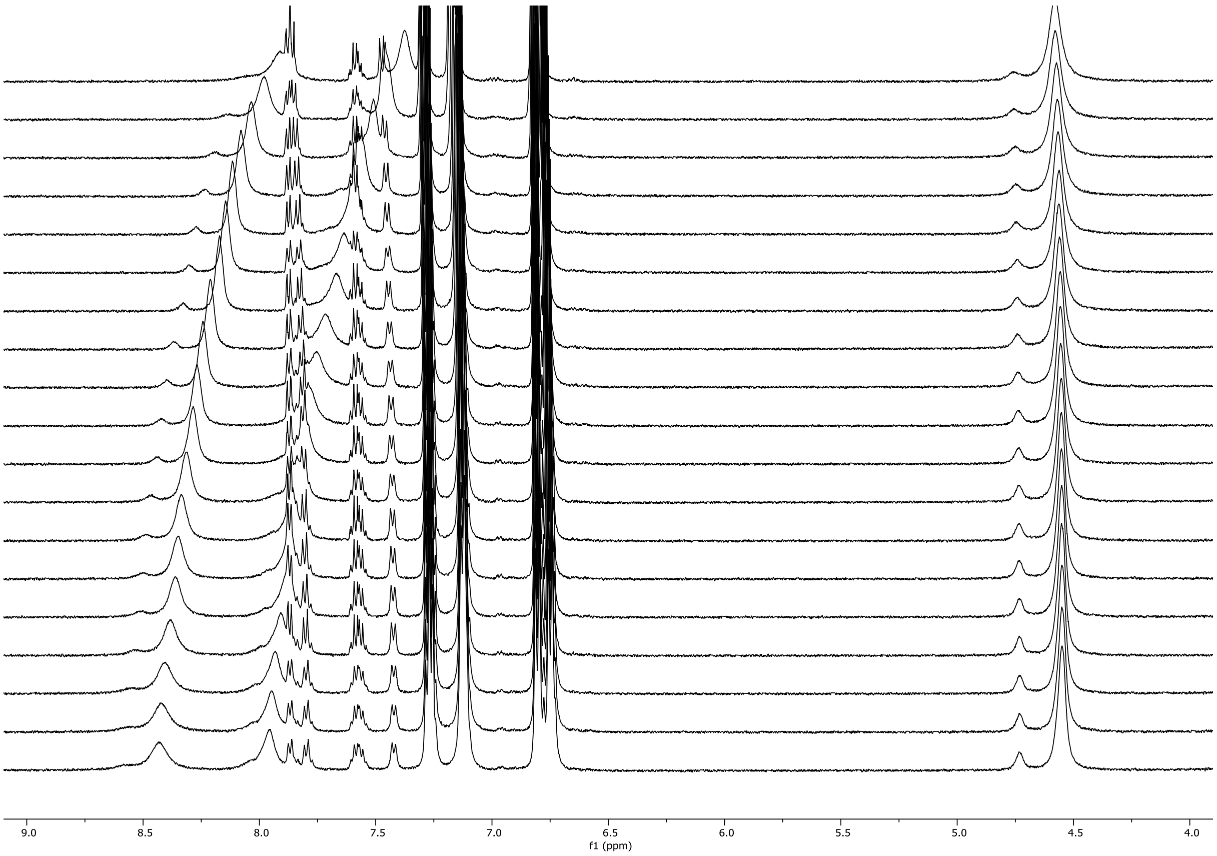

Figure S22. ^{1}H NMR spectral changes (500 MHz, 294 K) in the aromatic region of (Z_{PSS})-**2** in DMSO-d_6/0.5%H_2O (5.0×10^{-3} M) upon the stepwise addition of $[Bu_4N]^+[Cl]^-$ (from top to bottom: 0.00, 0.25, 0.50, 0.74, 0.97, 1.20, 1.43, 1.87, 2.30, 2.71, 3.11, 3.86, 4.56, 5.22, 5.84, 7.23, 9.48, 11.24, 12.70 equivalents).

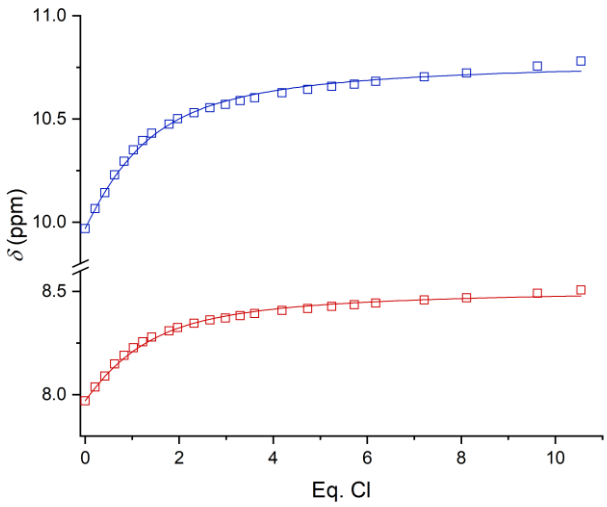

Figure S23. Titration curve for the addition of $[Bu_4N]^+[Cl]^-$ to (*E*,*E*,*E*)-**1** and data fit obtained by simultaneous analysis of the NH signals; $K_a = 2.7\times10^2$ M^{-1}.

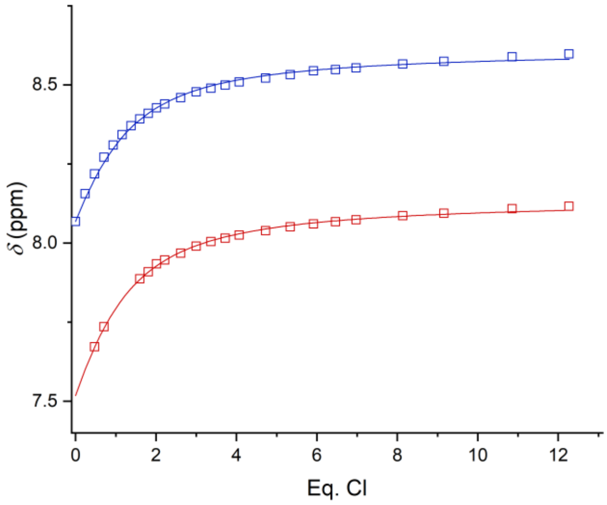

Figure S24. Titration curve for the addition of $[Bu_4N]^+[Cl]^-$ to (*E*,*E*,*E*)-**2** and data fit obtained by simultaneous analysis of the NH signals; $K_a = 2.9\times10^2$ M^{-1}.

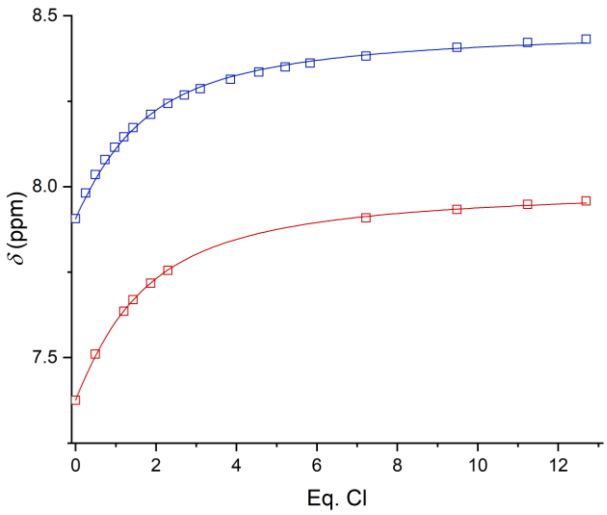

Figure S25. Titration curve for the addition of $[Bu_4N]^+[Cl]^-$ to (Z_{PSS})-**2** and data fit obtained by simultaneous analysis of the NH signals; $K_a = 1.8\times10^2$ M^{-1}.

HPLC separation of isomers

Part of a stock solution of (*E*,*E*,*E*)-**2** in DMSO/MeCN (1:1 *v*/*v*, 2 mM) was diluted with H_2O/MeCN (1:1 *v*/*v*) to reach a concentration of 80 µM. Subsequently it was allowed to equilibrate to the dark-adapted isomer ratio. The remaining stock solution was irradiated at 365 nm until the PSS was reached, affording the (Z_{PSS})-isomers, as was confirmed by UV/Vis spectroscopy. It was then also diluted with H_2O/MeCN (1:1 *v*/*v*) to a concentration of 80 µM. Both samples (20 µl) were injected on a C18-column (see the experimental section for details) and the isocratic method was used with an eluent containing 70% MeCN and 30% H_2O at a flow rate of 1.0 ml/min. The individual isomers were identified by recording a UV/Vis spectrum of each fraction, and the absorbances were normalized by setting the absorption value at the isosbestic point at 276 nm equal (see Figure S27).

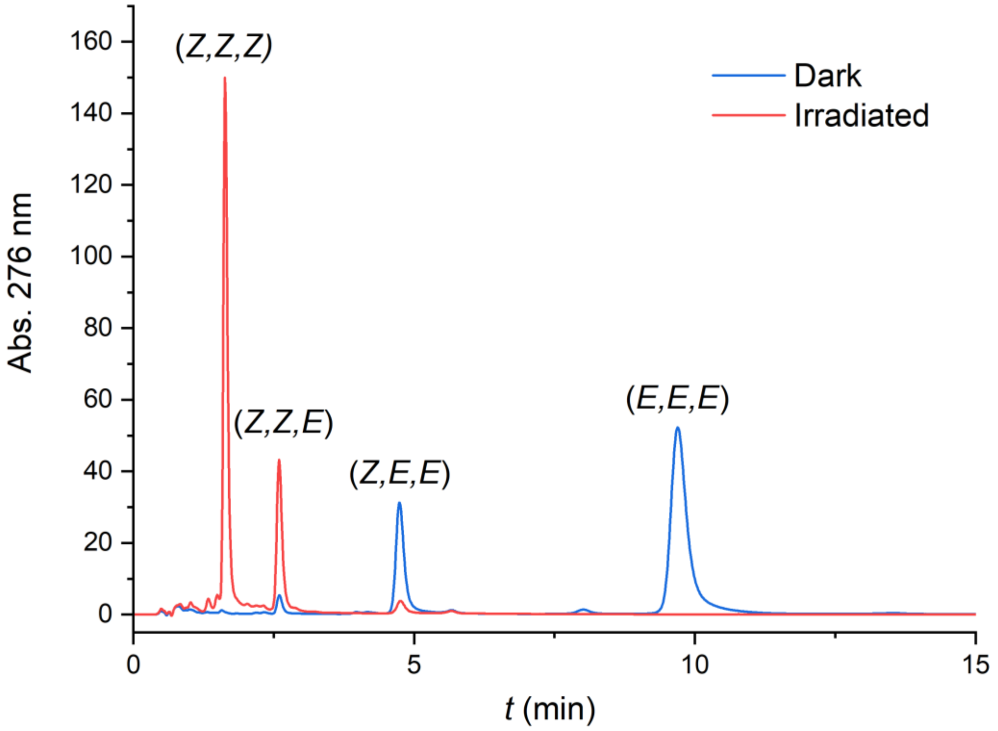

Figure S26. HPLC chromatogram of dark adapted **2** (blue) and (Z_{PSS})-**2** (red), showing separation of the individual isomers; UV detection was performed at 276 nm.

Table S1. Retention time of injected isomers.

	Z,Z,Z	*Z,Z,E*	*Z,E,E*	*E,E,E*
t_R (min)	1.63	2.59	4.74	9.69

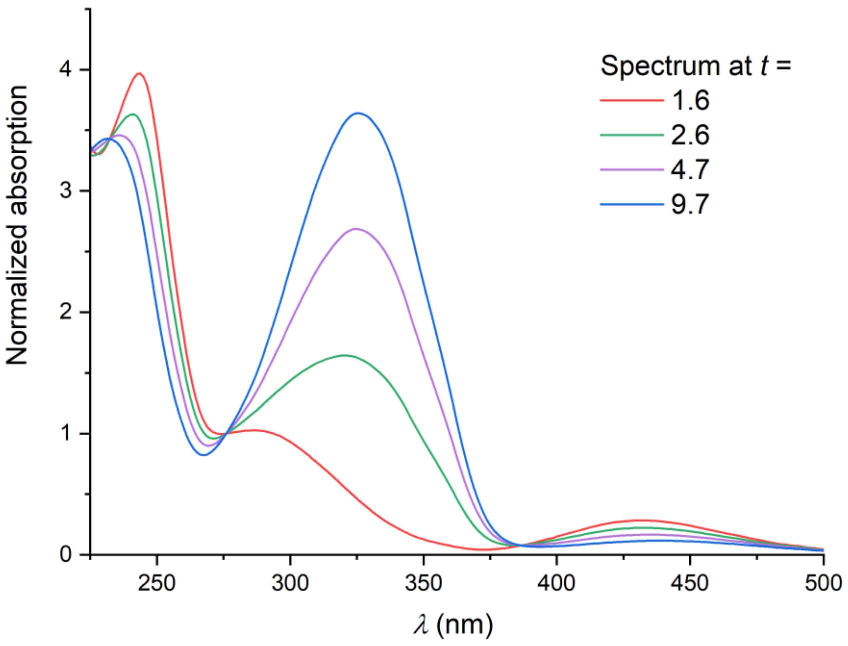

Figure S27. Normalized UV-Vis spectra recorded for the fractions that were isolated by HPLC.

Transmembrane transport experiments

Vesicle preparation – HPTS assay

Transport assays were performed using POPC vesicles loaded with the pH-responsive fluorescent dye HPTS. A 10 mM POPC solution in $CHCl_3$ was prepared in a round-bottom flask and the solvent was evaporated to create a lipid film, which was dried in vacuo for at least 12 hours. The lipid film was hydrated by vortexing with the internal solution, containing HPTS (1 mM) and NaCl (100 mM) buffered to pH 7.0 with HEPES (10 mM). Subsequently the suspension was subjected to 9 freeze and thaw cycles, by freezing in a liquid nitrogen bath, followed by thawing in a water bath of 45°C. After standing at room temperature for 30 minutes, the suspension was extruded 25 times through a 200 nm polycarbonate membrane to obtain unilamellar vesicles. Non-encapsulated dye was removed on an Illustra NAP™-25 Sephadex® G-25 column using the external solution containing NaCl (100 mM) buffered to pH 7.0 with HEPES (10 mM), affording a stock solution with a known concentration.

For each measurement, the stock solution was diluted with buffer to a standard volume (2.5 mL) in a PS cuvette to obtain a solution with a concentration of 0.1 mM in lipids. The sample was stirred at room temperature in the fluorimeter, and the fluorescence ratio of HPTS (λ_{ex} = 454 nm, λ_{em} = 511 nm, base form, divided by λ_{ex} = 403 nm, λ_{ex} = 511 nm, acid form) was measured over time. At t = 30 s the compounds were added as a DMSO/MeCN solution (5 µL, varying in concentration diluted from a 5 mM stock solution). To initiate transport, a pulse of NaOH (25 µL, 0.5 M) was given at t = 60 s to generate a pH gradient of pH 7 inside and pH 8 outside. Vesicles were lysed with Triton X-100 (50 µL, 11 wt% in H_2O/DMSO 7:1 *v*/*v*) at t = 370 s, and a final reading was taken at t = 430 s. The fractional fluorescent intensity (I_f) was then calculated using the following formula:

$$I_f = \frac{R_t - R_0}{R_d - R_0} \quad (\mathbf{S1})$$

Where R_t is the fluorescent ratio at time t, R_0 is the initial fluorescent ratio measured just prior to the base-pulse at t = 59 s, and R_d is the final ratio measured at t = 430 s.

To determine the activity of the (Z_{PSS})-isomers a stock solution of the (*E*,*E*,*E*)-isomer in DMSO/MeCN (1:1 *v*/*v*, 5 mM) was irradiated to PSS as indicated by UV/Vis spectroscopy. The stock solution of transporter **1** was irradiated continuously to maintain the PSS mixture as the thermal back isomerization was fast. Immediately prior to the run this solution was diluted to the desired concentration.

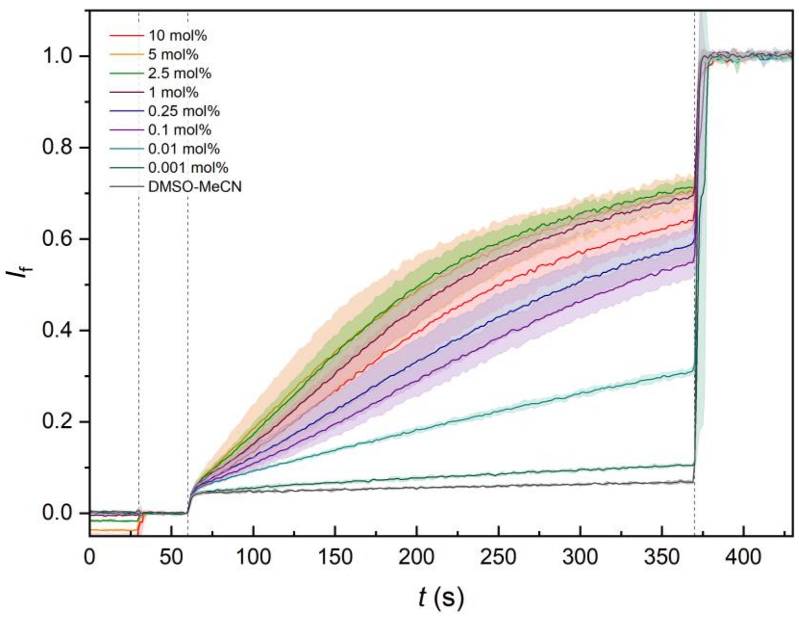

Figure S28. Plots of H^+/Cl^- symport (or Cl^-/OH^- antiport) against time across a POPC membrane facilitated by (*E*,*E*,*E*)-**1** (each measurement done in triplicate). The dashed lines at 30 s, 60 s and 370 s denote the moments the transporter is added from a DMSO/MeCN (1:1 *v*/*v*) solution, addition of the base pulse, and the lysis of the vesicles by addition of triton X-100, respectively.

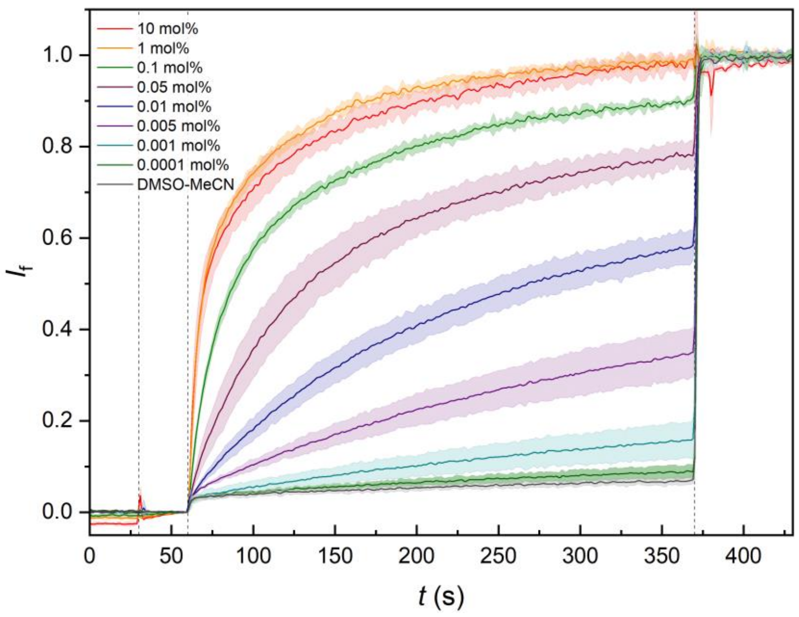

Figure S29. Plots of H^+/Cl^- symport (or Cl^-/OH^- antiport) against time across a POPC membrane facilitated by (Z_{PSS})-**1** (each measurement done in triplicate). The dashed lines at 30 s, 60 s and 370 s denote the moments the transporter is added from a DMSO/MeCN (1:1 *v*/*v*) solution, addition of the base pulse, and the lysis of the vesicles by addition of triton-X-100, respectively.

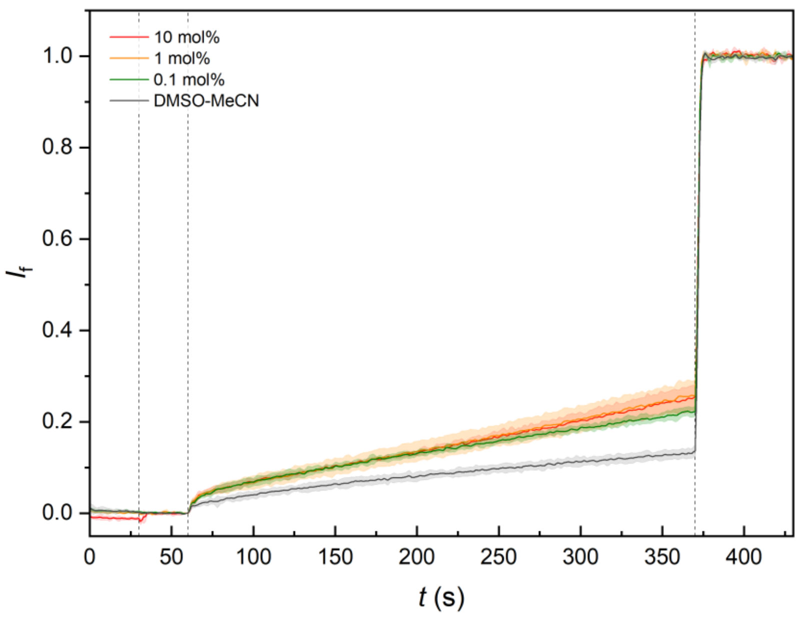

Figure S30. Plots of H^+/Cl^- symport (or Cl^-/OH^- antiport) against time across a POPC membrane facilitated by (*E*,*E*,*E*)-**2** (each measurement done in triplicate). The dashed lines at 30 s, 60 s and 370 s denote the moments the transporter is added from a DMSO/MeCN (1:1 *v*/*v*) solution, addition of the base pulse, and the lysis of the vesicles by addition of triton-X-100, respectively.

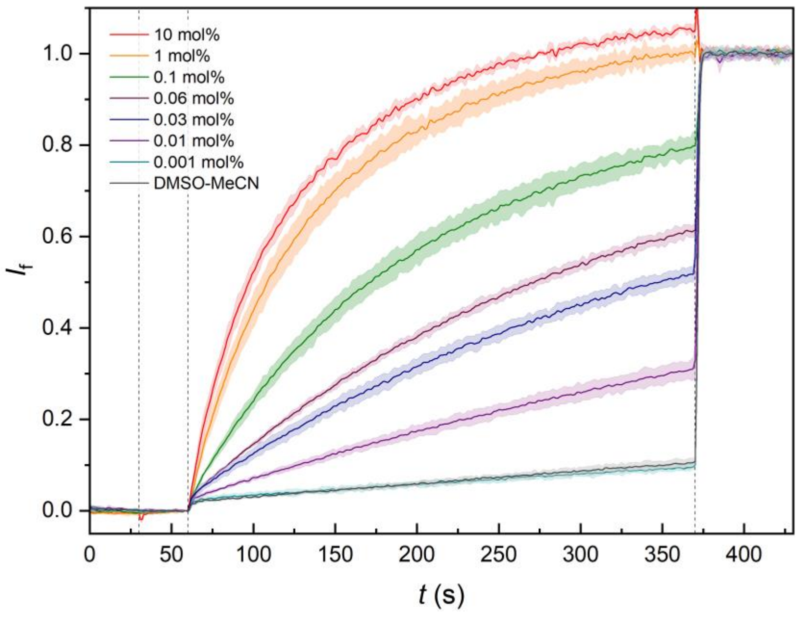

Figure S31. Plots of H^+/Cl^- symport (or Cl^-/OH^- antiport) against time across a POPC membrane facilitated by (Z_{PSS})-**2** (each measurement done in triplicate). The dashed lines at 30 s, 60 s and 370 s denote the moments the transporter is added from a DMSO/MeCN (1:1 *v*/*v*) solution, addition of the base pulse, and the lysis of the vesicles by addition of triton-X-100, respectively.

Hill analysis – HPTS assay

The fractional fluorescent intensity measured at t = 360 s was plotted as a function of transporter concentration. Using Origin 2022 this data was fitted to the Hill equation:

$$y = y_0 + (y_{max} - y_0)\frac{x^n}{k^n + x^n} \quad (\mathbf{S2})$$

Where y is the I_f at t = 360 s and x is the transporter concentration (in mol% with respect to lipids). The parameters to be fitted are: y_0 which is the I_f when no transporter is added, y_{max} is the maximum I_f that is obtained by the receptor, k is the concentration needed to get 50% of the maximum I_f and n is the Hill coefficient. The EC_{50} value is defined as the concentration needed to get 50% of the maximum possible chloride efflux, and is not represented by the k value when transporters have an incomplete Hill plot. To correctly assign the true EC_{50} value, the following equation was used:

$$EC_{50} = k\left(\frac{0.5 - 0.5y_0}{y_{max} - 0.5 - 0.5y_0}\right)^{1/n} \quad (\mathbf{S3})$$

The result of the Hill analysis is shown in the legend for each assay.

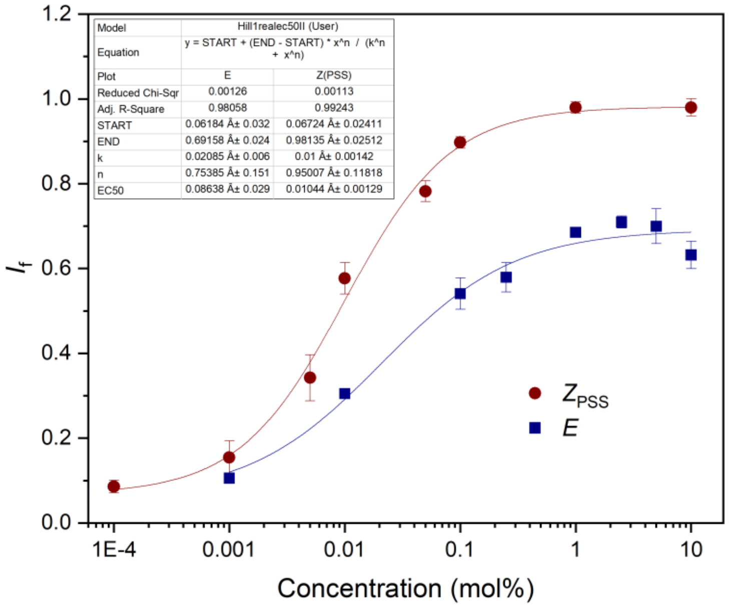

Figure S32. Plot of the fractional fluorescent intensity at 360 s in the HPTS assay against concentration facilitated by (*E*,*E*,*E*)-**1** (blue) and (Z_{PSS})-**1** (red), and the fit to the Hill equation. The same data is shown in the main text without the details of the fit.

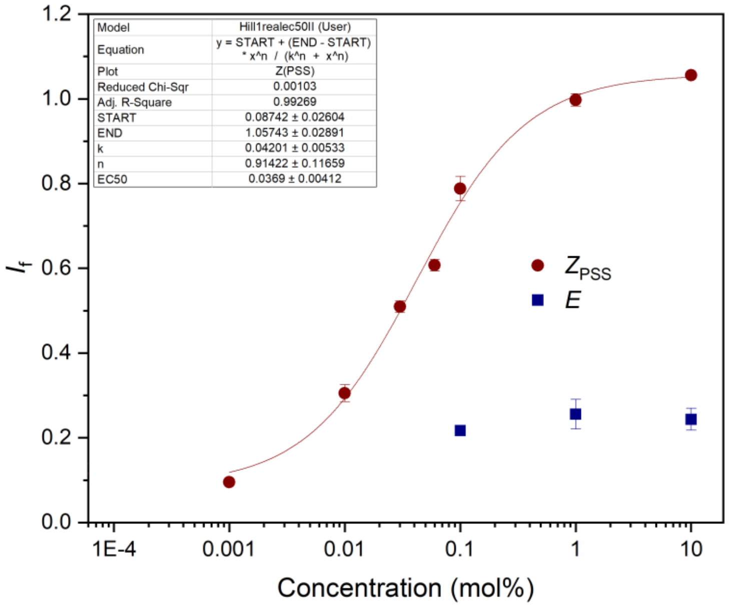

Figure S33. Plot of the fractional fluorescent intensity at 360 s in the HPTS assay against concentration facilitated by (*E*,*E*,*E*)-**2** (blue) and (Z_{PSS})-**2** (red), and the fit to the Hill equation. The same data is shown in the main text without the details of the fit.

In situ irradiation – HPTS assay

A vesicle solution (0.1 mM in lipids, 2.5 mL) was prepared according to the above protocol for the HPTS assay. The fluorescence ratio of HPTS was followed over time, and converted to the fractional fluorescent intensity (I_f) using equation **S1**. At t = 30 s the compounds were added as a DMSO/MeCN (1:1 *v*/*v*) solution (5 µL, varying in concentration diluted from a 5 mM stock solution). At t = 60 s, the NaOH base-pulse (25 µL, 0.5 M) was added to initiate transport. During the experiment samples were irradiated between t = 120 – 150 s using a 385 nm or 365 nm LED mounted on the lid of the fluorimeter. After t = 370 s vesicles were lysed with Triton X-100 (50 µL, 11 wt% in H_2O/DMSO 7:1 *v*/*v*), and a final reading was taken at t = 430 s. The traces of the irradiated samples were compared to traces of the samples that were not irradiated.

Vesicle preparation – Osmotic assay

For the osmotic assay, vesicles with transporter pre-incorporated were prepared from a stock solution of 10 mM POPC and 1 mM transporter (10 mol%) in $CHCl_3$ that was diluted to the appropriate molar ratio with a 10 mM POPC solution and added to a round bottom flask. In case no transporter was pre-incorporated, only a 10 mM POPC solution was added to the flask. The solvent was evaporated to create a lipid film, which was dried in vacuo for at least 12 hours. The film was hydrated by vortexing with the internal solution consisting of KCl (300 mM) buffered to pH 7.20 with HEPES (10 mM). Next, the suspension was subjected to 9 freeze and thaw cycles, using liquid nitrogen and a water bath of 45°C. The solution was left standing at room temperature for 30 minutes, and subsequently extruded 25 times through a 400 nm polycarbonate membrane to obtain unilamellar vesicles. The external components were removed by an Illustra NAP™-25 Sephadex® G-25 column, and exchanged for the external solution containing KGlu (300 mM) buffered to pH 7.20 with HEPES (10 mM), which afforded stock solutions of a known concentration.

For each measurement, the stock solution was diluted with buffer to a standard volume (2.2 mL) in a PS cuvette to obtain a solution with a concentration of 0.4 mM in lipids. The sample was stirred at room temperature in the fluorimeter, and the 90° light scattering intensity (λ_{ex} = 600 nm, λ_{em} = 600 nm) was measured over time. When the transporter was not pre-incorporated it was added at t = 30 s as a DMSO/MeCN (1:1 *v*/*v*) solution (5 µL, varying in concentration diluted from a 5 mM stock solution). At t = 60 s a DMSO solution of valinomycin or monensin (5 µL, 0.176 mM, 0.1 mol%) was added to initiate transport. Between t = 660 – 670 s prodigiosin (5 µL, 17.6 µM, 0.01 mol%) and FCCP (5 µL, 1.76 mM, 1 mol%) were added to reach full efflux of the remaining chloride, and a final reading corresponding to 100% efflux was taken at t = 1260 s. When monensin was used as cotransporter only prodigiosin was added. The fractional light scattering intensity (I_f) was then calculated using the following formula:

$$I_f = \frac{I_t - I_0}{I_l - I_0} \quad (\mathbf{S4})$$

Where I_t is the light scattering intensity at time t, I_0 is the initial light scattering intensity measured just prior to the addition of the cotransporter calculated as the average scattering between t = 48 – 58 s, and I_l is the light scattering intensity after all chloride is transported calculated as the average scattering between t = 1250 – 1260 s.

To determine the activity of the (Z_{PSS})-isomers after post addition, a stock solution of the (*E,E,E)*-isomer of **1** and **2** in DMSO/MeCN (1:1 *v*/*v*, 5 mM) was irradiated to PSS as indicated by UV/Vis spectroscopy. The stock solution of transporter **1** was irradiated continuously to maintain the PSS mixture as the thermal back isomerization was fast. Immediately prior to the run this solution was diluted to the desired concentration. To maintain the PSS mixture of (Z_{PSS})-**1** after addition, samples were also continuously irradiated during the assay. Irradiation was stopped at the point where prodigiosin (and FCCP) were added. During measurements with (Z_{PSS})-**2** irradiation of the transporter was not necessary as here thermal back isomerization is slow.

To determine the activity of the (Z_{PSS})-isomers when incorporated, a sample with the same vesicles that were used to determine the activity of the pre-incorporated (*E*,*E*,*E*)-isomer were irradiated to PSS immediately prior to the start of the assay. Transporter **1** was irradiated for 1 minute with 385 nm light, after which it was continuously irradiated during the assay to maintain the PSS mixture since thermal back isomerization is fast. Irradiation was stopped at the point where prodigiosin (and FCCP) were added. For transporter **2** irradiation to PSS took 2 minutes for concentrations of 0.1 mol% and lower, and 4 minutes for concentrations up to 3 mol%, as determined by UV-Vis measurements. During the assay irradiation of the transporter was not necessary as here thermal back isomerization is slow.

Integrity of irradiated vesicles

To study any additional undesired effects of irradiation during the osmotic assay, vesicles were prepared with transporter pre-incorporated at the highest concentration used for Hill analysis as described above. Samples were measured as usual with the modification that no cotransporter was added to initiate transport. To demonstrate that the integrity of the vesicle was not compromised, chloride efflux was triggered between *t* = 660 – 670 s by the addition of valinomycin (5 µL, 0.176 mM, 0.1 mol%), prodigiosin (5 µL, 17.6 µM, 0.01 mol%) and FCCP (5 µL, 1.76 mM, 1 mol%). Samples were continuously irradiated with 365 nm or 385 nm from the start of the assay until the point where valinomycin, prodigiosin and FCCP were added. The traces of irradiated samples were compared to traces that were not irradiated.

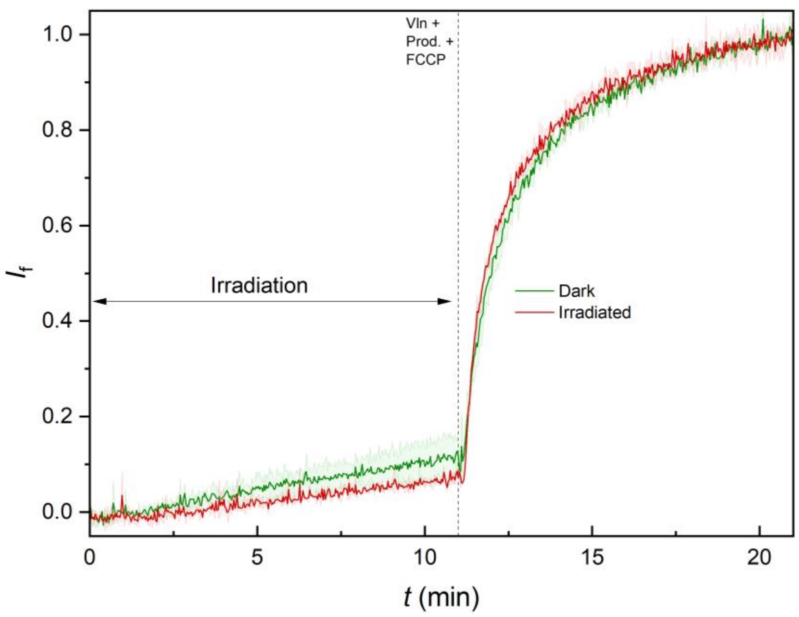

Figure S34. Plots of the effect of irradiation on the integrity of vesicle incorporating 1 mol% (*E*,*E*,*E*)-**1** during the osmotic assay. The dashed line at 11 minutes denotes the moment of addition of valinomycin, prodigiosin and FCCP.

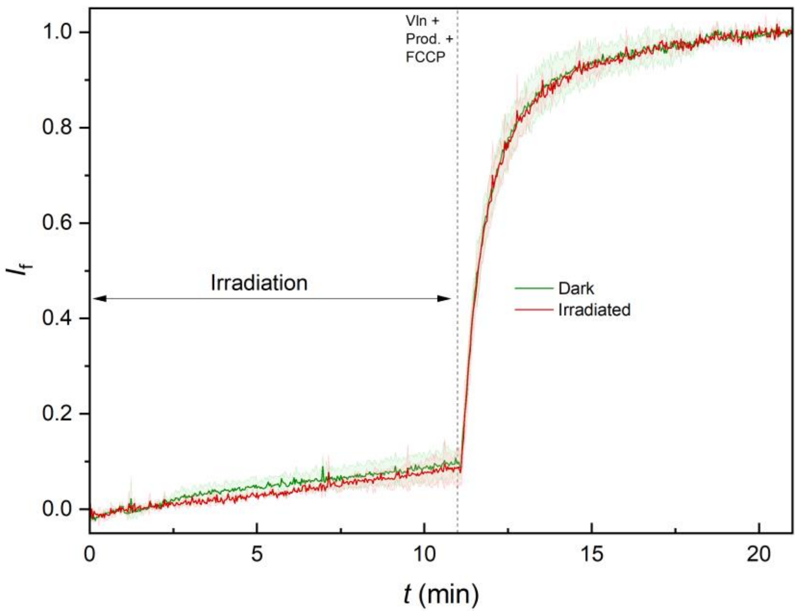

Figure S35. Plots of the effect of irradiation on the integrity of vesicle incorporating 3 mol% (*E*,*E*,*E*)-**2** during the osmotic assay. The dashed line at 11 minutes denotes the moment of addition of valinomycin, prodigiosin and FCCP.

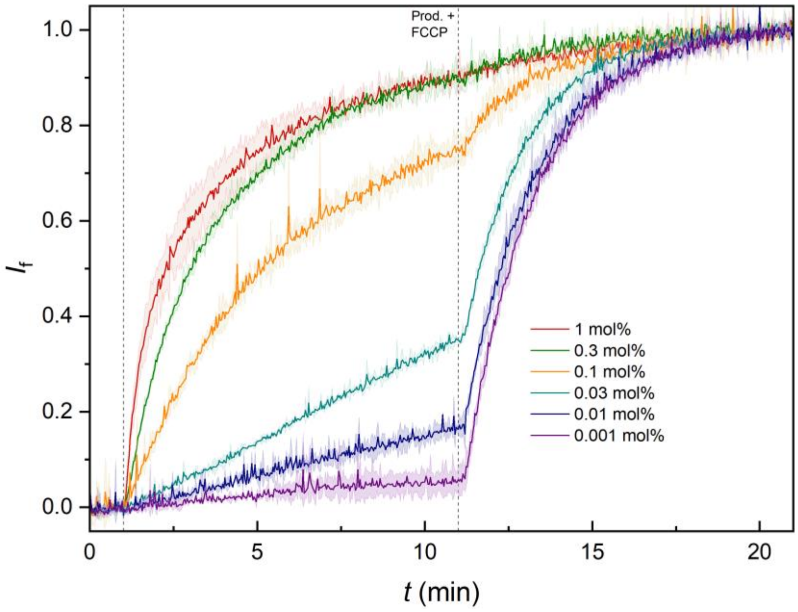

Figure S36. Plots of electrogenic $Cl^−$ transport mediated by pre-incorporated (*E*,*E*,*E*)-**1** in combination with valinomycin against time across a POPC membrane (each measurement done in duplicate). The dashed lines at 1 and 11 minutes denote the moments where valinomycin is added, and the addition of prodigiosin and FCCP, respectively.

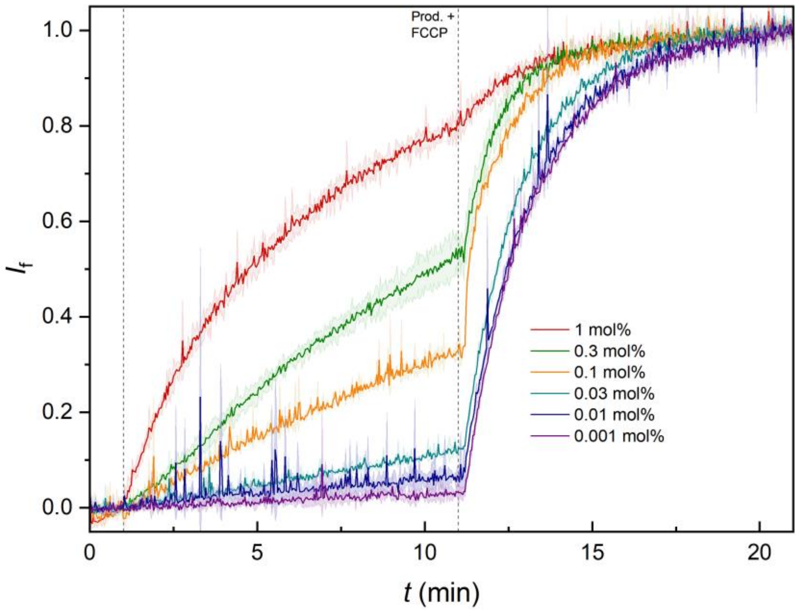

Figure S37. Plots of electrogenic $Cl^−$ transport mediated by pre-incorporated (Z_{PSS})-**1** in combination with valinomycin against time across a POPC membrane (each measurement done in duplicate). The dashed lines at 1 and 11 minutes denote the moments where valinomycin is added, and the addition of prodigiosin and FCCP, respectively.

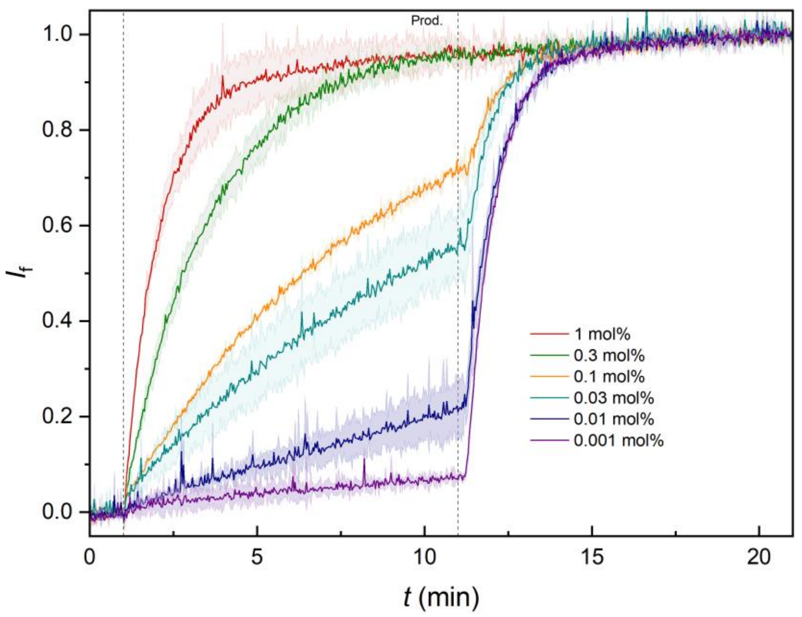

Figure S38. Plots of electroneutral Cl^- transport mediated by pre-incorporated (*E*,*E*,*E*)-**1** in combination with monensin against time across a POPC membrane (each measurement done in duplicate). The dashed lines at 1 and 11 minutes denote the moments where monensin is added, and the addition of prodigiosin, respectively.

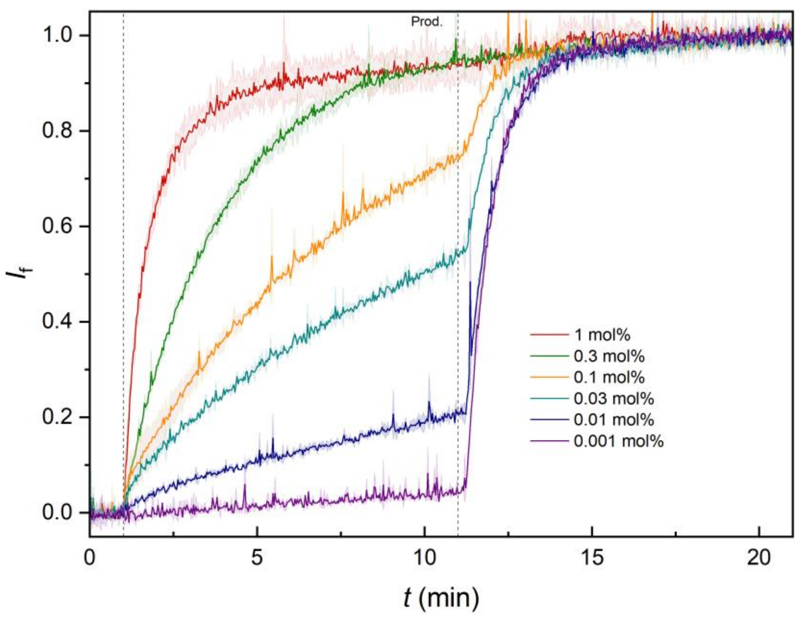

Figure S39. Plots of electroneutral Cl^- transport mediated by pre-incorporated (Z_{PSS})-**1** in combination with monensin against time across a POPC membrane (each measurement done in duplicate). The dashed lines at 1 and 11 minutes denote the moments where monensin is added, and the addition of prodigiosin, respectively.

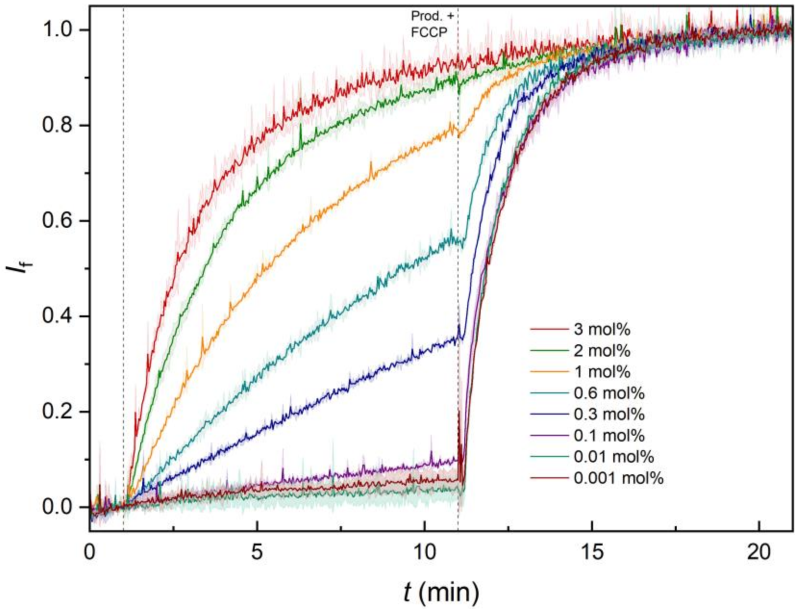

Figure S40. Plots of electrogenic Cl^- transport mediated by pre-incorporated (*E*,*E*,*E*)-**2** in combination with valinomycin against time across a POPC membrane (each measurement done in duplicate). The dashed lines at 1 and 11 minutes denote the moments where valinomycin is added, and the addition of prodigiosin and FCCP, respectively.

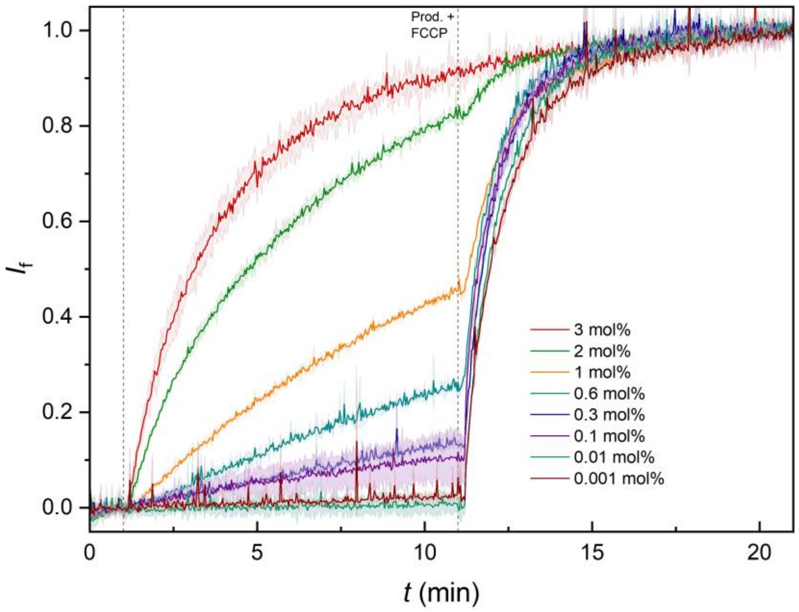

Figure S41. Plots of electrogenic Cl^- transport mediated by pre-incorporated (Z_{PSS})-**2** in combination with valinomycin against time across a POPC membrane (each measurement done in duplicate). The dashed lines at 1 and 11 minutes denote the moments where valinomycin is added, and the addition of prodigiosin and FCCP, respectively.

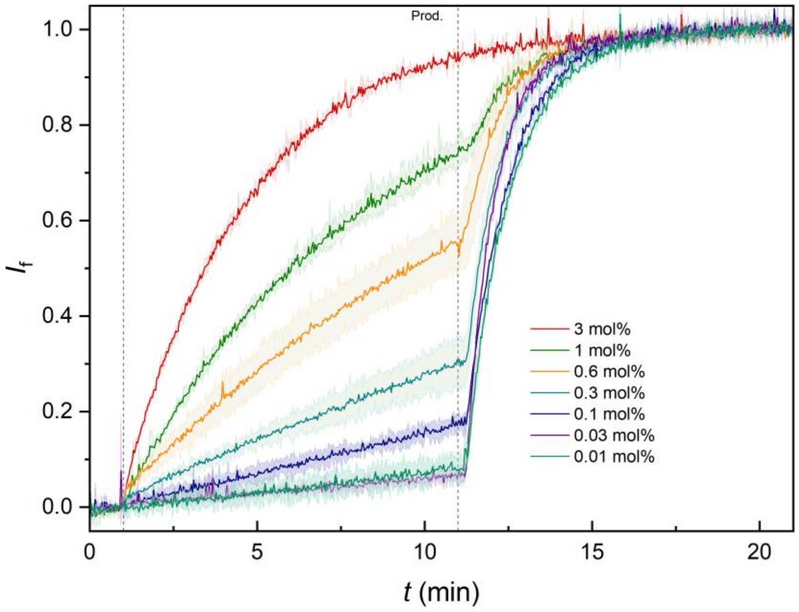

Figure S42. Plots of electroneutral Cl^- transport mediated by pre-incorporated (*E*,*E*,*E*)-**2** in combination with monensin against time across a POPC membrane (each measurement done in duplicate). The dashed lines at 1 and 11 minutes denote the moments where monensin is added, and the addition of prodigiosin, respectively.

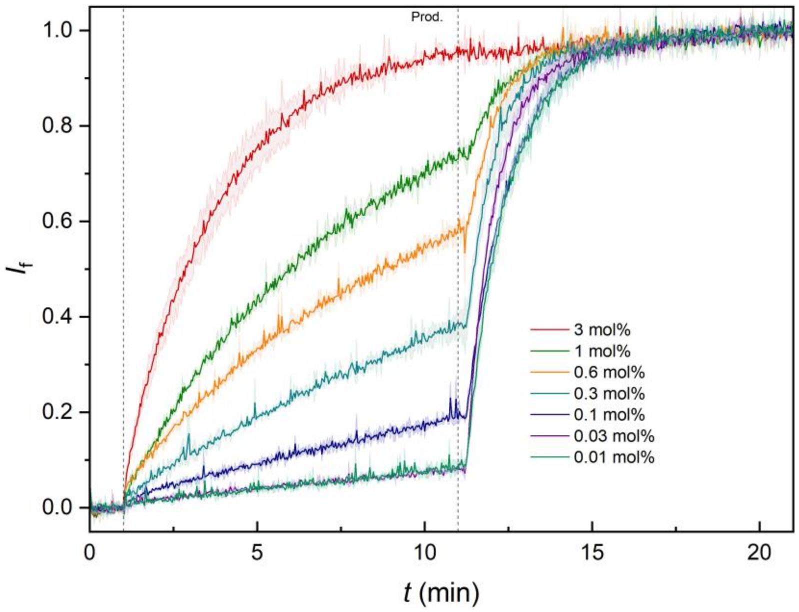

Figure S43. Plots of electroneutral Cl^- transport mediated by pre-incorporated (Z_{PSS})-**2** in combination with monensin against time across a POPC membrane (each measurement done in duplicate). The dashed lines at 1 and 11 minutes denote the moments where monensin is added, and the addition of prodigiosin, respectively.

Hill analysis – Osmotic assay

The fractional scattering intensity measured at t = 658 s was plotted as a function of transporter concentration. Using Origin 2022 this data was fitted to equation **S2 and S3**

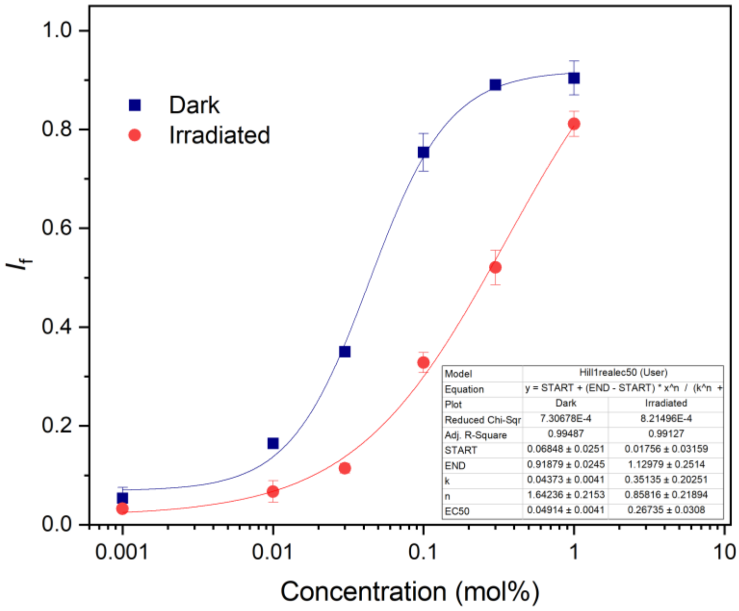

Figure S44. Plot of the fractional scattering intensity at 658 s in the electrogenic osmotic assay against concentration facilitated by (*E*,*E*,*E*)-**1** (blue) and (Z_{PSS})-**1** (red) in combination with valinomycin, and the fit to the Hill equation. The same data is shown in the main text without the details of the fit.

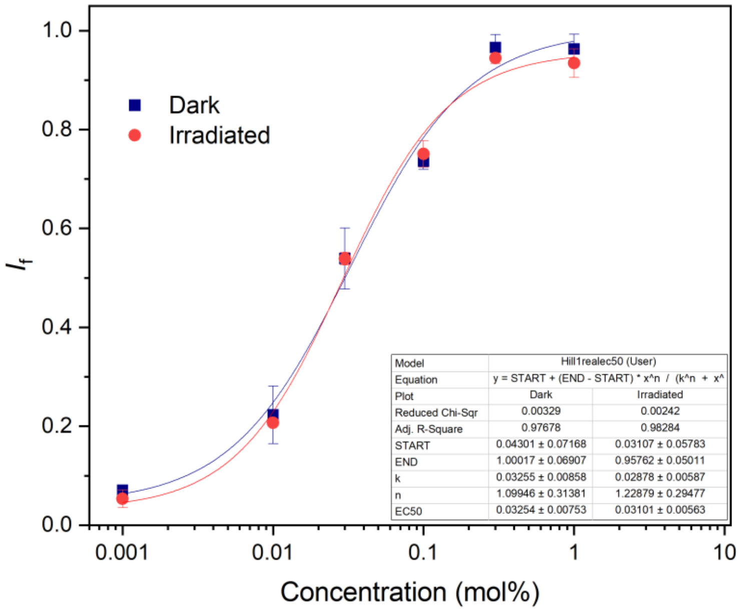

Figure S45. Plot of the fractional scattering intensity at 658 s in the electrogenic osmotic assay against concentration facilitated by (*E*,*E*,*E*)-**1** (blue) and (Z_{PSS})-**1** (red) in combination with monensin, and the fit to the Hill equation. The same data is shown in the main text without the details of the fit.

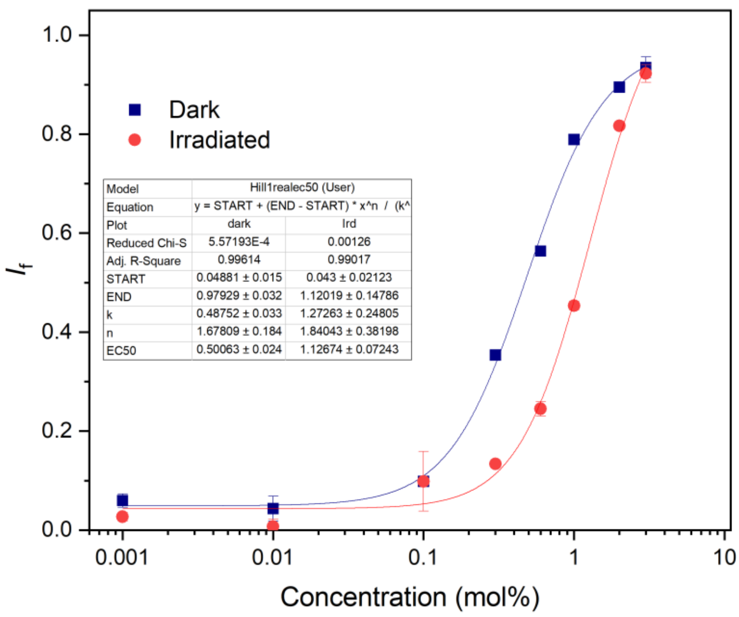

Figure S46. Plot of the fractional scattering intensity at 658 s in the electroneutral osmotic assay against concentration facilitated by (*E*,*E*,*E*)-**2** (blue) and (Z_{PSS})-**2** (red) in combination with valinomycin, and the fit to the Hill equation. The same data is shown in the main text without the details of the fit.

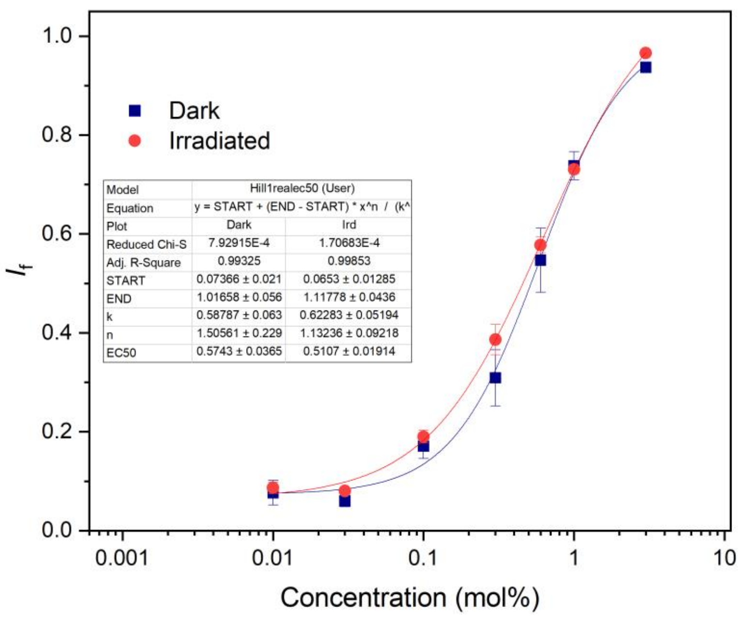

Figure S47. Plot of the fractional scattering intensity at 658 s in the electroneutral osmotic assay against concentration facilitated by (*E*,*E*,*E*)-**2** (blue) and (Z_{PSS})-**2** (red) in combination with monensin, and the fit to the Hill equation. The same data is shown in the main text without the details of the fit.

Effect of BSA and oleic acid – Osmotic assay

Vesicles with the transporter pre-incorporated were prepared according to the above protocol for the osmotic assay. To investigate the influence of fatty acids on the transport process, transport activity in these vesicles was compared after removal or addition of fatty acids. To remove fatty acids, the vesicles were treated with bovine serum albumin (BSA). This treatment was performed by diluting the vesicles with a BSA solution (1 mM) to obtain a mixture that is 5 mM in lipids and 50 μM in BSA (1 mol%), which was stirred for 30 minutes. To increase the fatty acids concentration of untreated vesicles, an oleic acid solution in buffer was added to the samples (5 μL, 8.8 mM, 5 mol%) immediately prior to the measurement. Traces were compared before and after BSA treatment, and after addition of oleic acid.

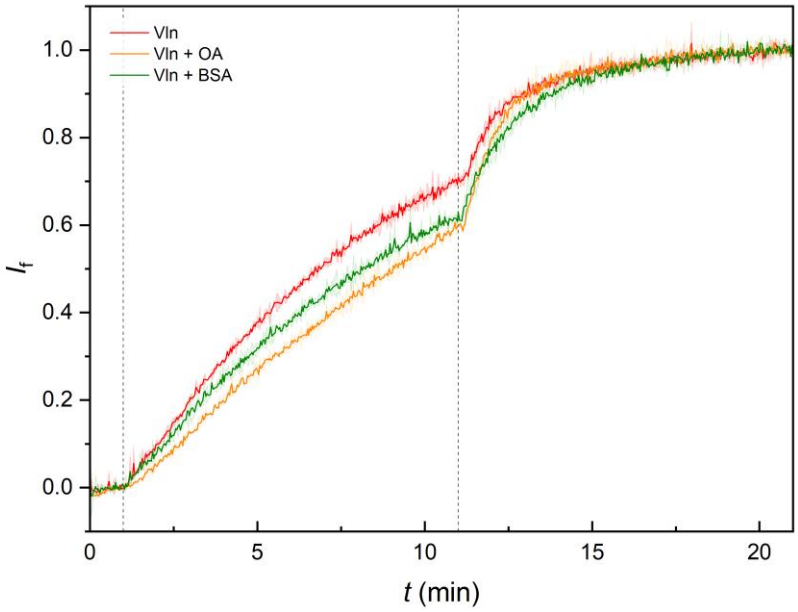

Figure S48. Plots of electrogenic Cl^- transport against time across a POPC membrane facilitated by 0.1 mol% (*E*,*E*,*E*)-**1** in combination with valinomycin, in vesicles with various amounts of fatty acids (each measurement done in duplicate). The dashed lines at 1 and 11 minutes denote the moments where valinomycin is added, and the addition of prodigiosin and FCCP, respectively.

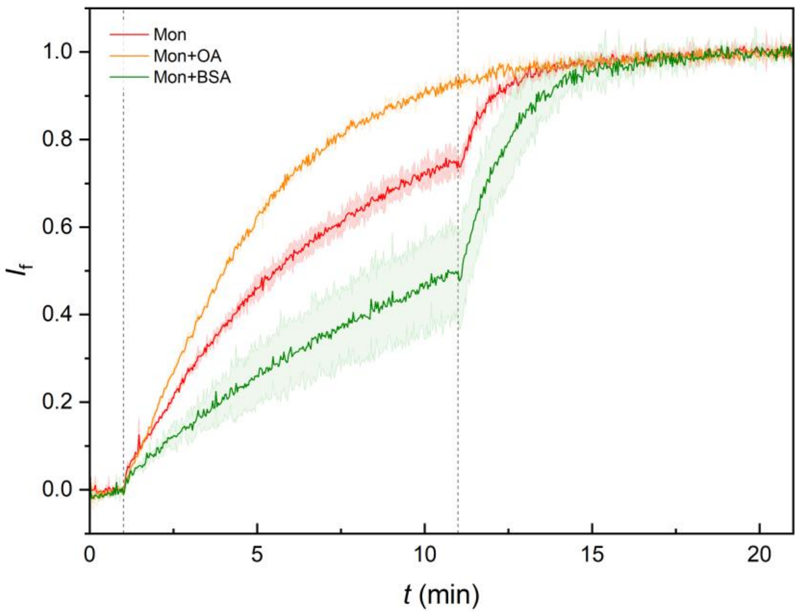

Figure S49. Plots of electroneutral H/Cl transport against time across a POPC membrane facilitated by 0.1 mol% (*E*,*E*,*E*)-**1** in combination with monensin, in vesicles with various amounts of fatty acids (each measurement done in duplicate). The dashed lines at 1 and 11 minutes denote the moments where monensin is added, and the addition of prodigiosin, respectively.

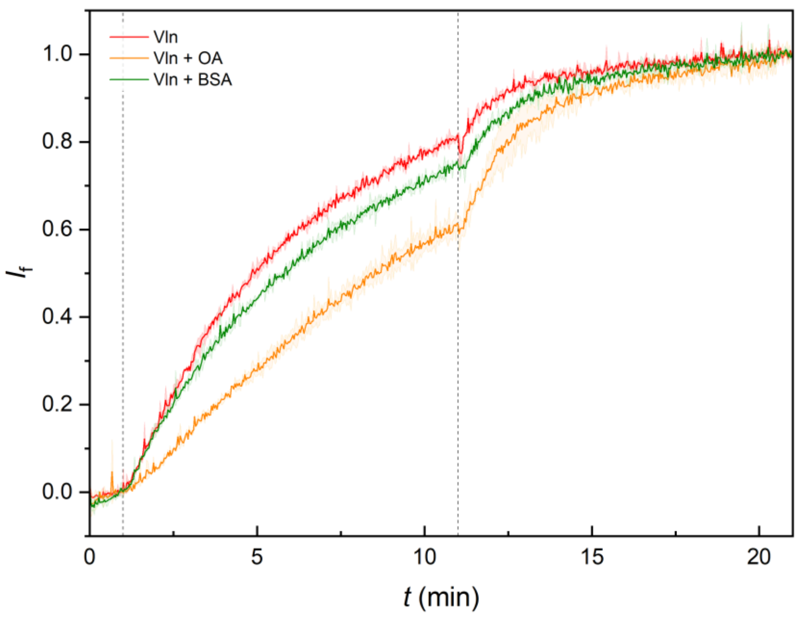

Figure S50. Plots of electrogenic Cl^- transport against time across a POPC membrane facilitated by 1 mol% (*E*,*E*,*E*)-**2** in combination with valinomycin, in vesicles with various amounts of fatty acids (each measurement done in duplicate). The dashed lines at 1 and 11 minutes denote the moments where valinomycin is added, and the addition of prodigiosin and FCCP, respectively.

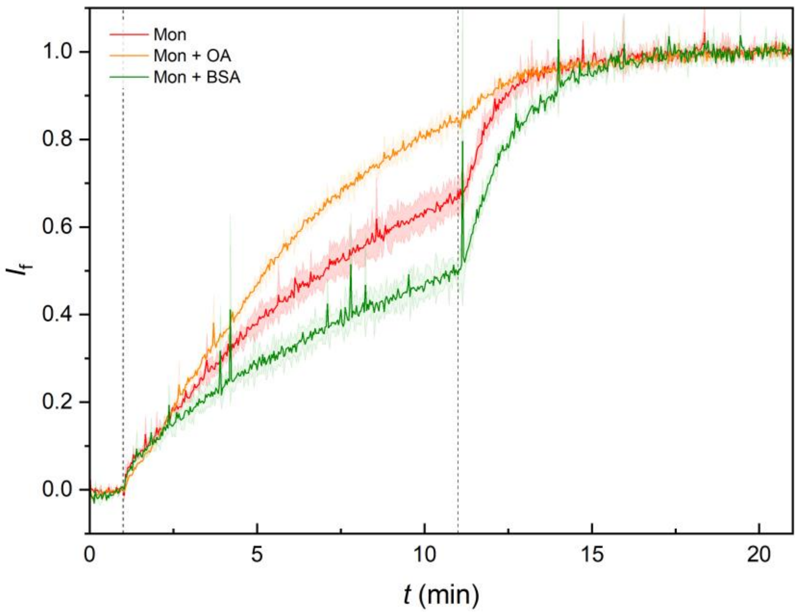

Figure S51. Plots of electroneutral H/Cl transport against time across a POPC membrane facilitated by 1 mol% (*E*,*E*,*E*)-**2** in combination with monensin, in vesicles with various amounts of fatty acids (each measurement done in duplicate). The dashed lines at 1 and 11 minutes denote the moments where monensin is added, and the addition of prodigiosin, respectively.

In situ irradiation – Osmotic assay

A vesicle solution (0.4 mM in lipids, 2.5 mL) was prepared as stated in the above protocol for the osmotic assay. Scattering intensity was followed over time, and converted to the fractional scattering intensity (I_f) using equation **S4**. At t = 30 s the compounds were added as a DMSO solution (5 µL, varying in concentration diluted from a 5 mM stock solution). At t = 60 s a DMSO solution of valinomycin (5 µL, 0.176 mM, 0.1 mol%) was added to initiate transport. During the experiment samples were irradiated at various times as indicated using 385 nm, 365 nm or 455 nm LEDs mounted on the lid of the fluorimeter. Between t = 3660 – 3670 s prodigiosin (5 µL, 17.6 µM, 0.01 mol%) and FCCP (5 µL, 1.76 mM, 1 mol%) were added to reach full efflux of the remaining chloride, and a final reading corresponding to 100% efflux was taken at t = 4260 s. The traces of the irradiated samples were compared to traces that were not irradiated, partially irradiated, and when no valinomycin was added.

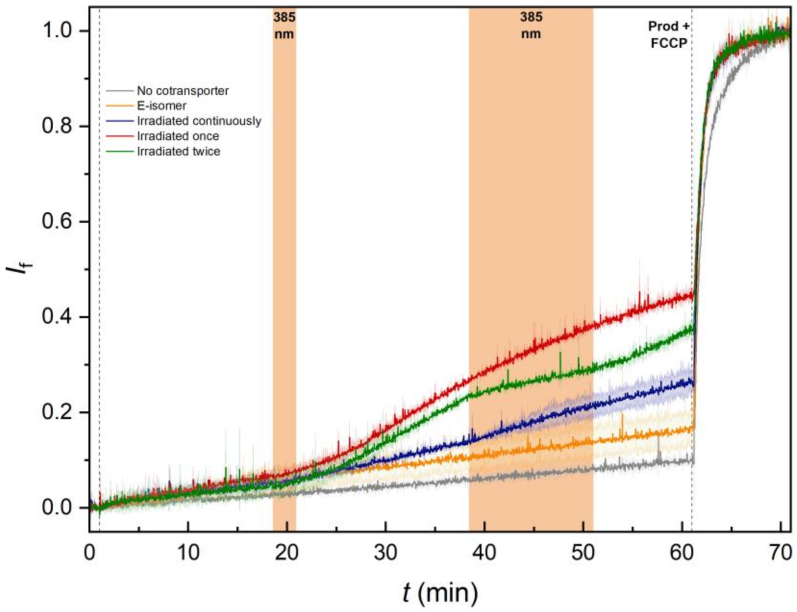

Figure S52. Plots of electrogenic Cl^- transport against time across a POPC membrane facilitated by 0.02 mol% (*E*,*E*,*E*)-**1** in combination with valinomycin, and activation by *in situ* irradiation with 385nm (each measurement done in duplicate).

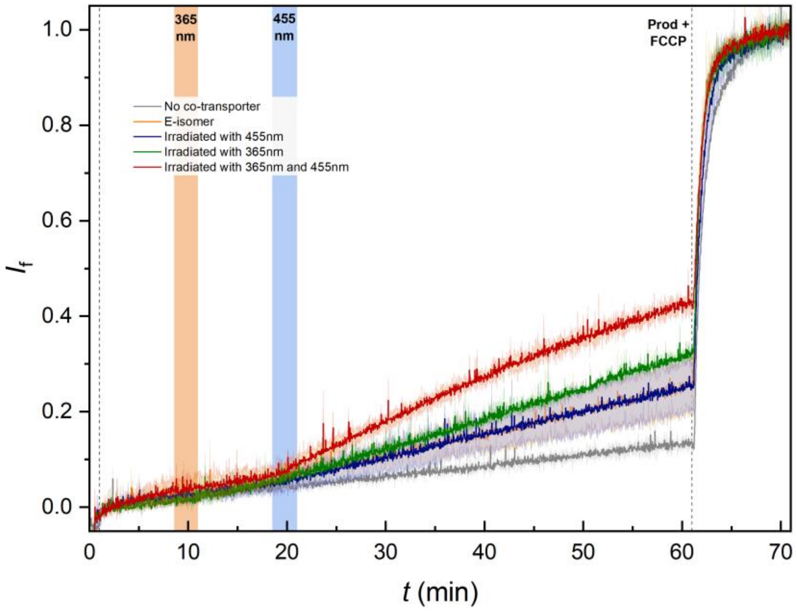

Figure S53. Plots of electrogenic Cl^- transport against time across a POPC membrane facilitated by 1 mol% dark adapted **2** in combination with valinomycin, and activation by *in situ* irradiation with 365 nm and 455 nm (each measurement done in duplicate).

Single crystal X-ray crystallography

All reflection intensities were measured at 110(2) K using a SuperNova diffractometer (equipped with Atlas detector) with Mo *K*α radiation (λ = 0.71073 Å) under the program CrysAlisPro (Version CrysAlisPro 1.171.39.29c, Rigaku OD, 2017). The same program was used to refine the cell dimensions and for data reduction. The structure was solved with the program SHELXS-2018/3 (Sheldrick, 2018) and was refined on F^2 with SHELXL-2018/3 (Sheldrick, 2018). Numerical absorption correction based on gaussian integration over a multifaceted crystal model was applied using CrysAlisPro. The temperature of the data collection was controlled using the system Cryojet (manufactured by Oxford Instruments). The H atoms were placed at calculated positions (unless otherwise specified) using the instructions AFIX 23 or AFIX 43 with isotropic displacement parameters having values 1.2 U_{eq} of the attached C atoms. The H atoms attached to N4, N7, N24, N27, N44 and N47 were found from difference Fourier maps, and their coordinates were refined pseudofreely using the DFIX instruction in order to keep the N–H distances within an acceptable range.

The structure is partly disordered. The phenyl group C36 → C41 and the fragment C48 → C61 are disordered over two orientations, and the occupancy factors of the major component of the disorder refine to 0.787(14) and 0.914(3), respectively.

Table S2. Experimental details

Crystal data	
Chemical formula	$C_{45}H_{45}N_{13}S_3$
M_r	864.12
Crystal system, space group	Monoclinic, $P2_1/c$
Temperature (K)	110
a, *b*, *c* (Å)	15.6936 (3), 9.73474 (19), 29.0803 (6)
β (°)	104.532 (2)
V (Å^3)	4300.56 (15)
Z	4
Radiation type	Mo *K*α
μ (mm^{-1})	0.22
Crystal size (mm)	0.30 × 0.22 × 0.07
Data collection	
Diffractometer	SuperNova, Dual, Cu at zero, Atlas
Absorption correction	Gaussian<br>*CrysAlis PRO* 1.171.39.29c (Rigaku Oxford Diffraction, 2017) Numerical absorption correction based on gaussian integration over a multifaceted crystal model Empirical absorption correction using spherical harmonics, implemented in SCALE3 ABSPACK scaling algorithm.
T_{min}, T_{max}	0.743, 1.000
No. of measured, independent and observed [$I > 2\sigma(I)$] reflections	63486, 9881, 8608
R_{int}	0.029
$(\sin \theta/\lambda)_{max}$ (Å^{-1})	0.650
Refinement	
$R[F^2 > 2\sigma(F^2)]$, $wR(F^2)$, S	0.035, 0.092, 1.05
No. of reflections	9881
No. of parameters	744
No. of restraints	713
H-atom treatment	H atoms treated by a mixture of independent and constrained refinement
$\Delta\rho_{max}$, $\Delta\rho_{min}$ (e Å^{-3})	0.30, -0.24

Computer programs: *CrysAlis PRO* 1.171.39.29c (Rigaku OD, 2017), *SHELXS2018*/3 (Sheldrick, 2018), *SHELXL2018*/3 (Sheldrick, 2018), *SHELXTL* v6.10 (Sheldrick, 2008).[6]

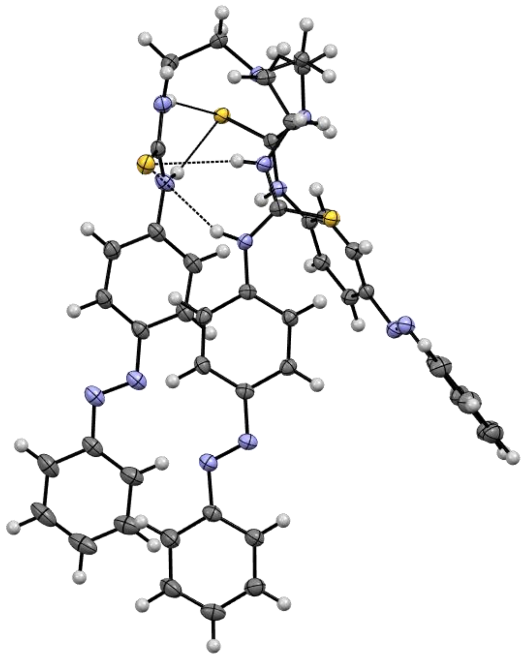

Figure S54. Displacement ellipsoid plot (50% probability level) of (*E,E,E*)-**1** found in the solid state at 110(2) K showing intermolecular NH···S hydrogen bonding interactions.

References

(1) Zhang, X.; Woggon, W.-D. *J. Am. Chem. Soc.* **2005**, *127*, 14138–14139.

(2) Rodríguez-Soacha, D. A.; Steinmüller, S. A. M.; Işbilir, A.; Fender, J.; Deventer, M. H.; Ramírez, Y. A.; Tutov, A.; Sotriffer, C.; Stove, C. P.; Lorenz, K.; et al. *ACS Chem. Neurosci.* **2022**, *13*, 2410–2435.

(3) Villarón, D.; Bos, J. E.; Kohl, F.; Mommer, S.; de Jong, J.; Wezenberg, S. J. *J. Org. Chem.* **2023**, 88, 11328–11334.

(4) Fulmer, G. R.; Miller, A. J. M.; Sherden, N. H.; Gottlieb, H. E.; Nudelman, A.; Stoltz, B. M.; Bercaw, J. E.; Goldberg, K. I. *Organometallics* **2010**, *29*, 2176–2179.

(5) Frassineti, C.; Ghelli, S.; Gans, P.; Sabatini, A.; Moruzzi, M. S.; Vacca, A. *Anal. Biochem.* **1995**, *231*, 374–382.

(6) Sheldrick, G. M. *Acta Crystallogr. Sect. C Struct. Chem.* **2015**, *71*, 3–8.
